# Crosstalk between strigolactone and KAI2/KL signalling in plant growth and stress responses

**DOI:** 10.3389/fpls.2026.1931114

**Published:** 2026-09-21

**Authors:** Ruihan Shen, Silvia Gianoglio, Ivan Visentin, Andrea Schubert, Francesca Cardinale

**Affiliations:** Department of Agricultural, Forest and Food Sciences (DISAFA), University of Turin, Grugliasco, Italy

**Keywords:** abiotic stress, crosstalk, karrikins, signalling, strigolactones

## Abstract

Strigolactones (SLs) and karrikins (KARs) are signalling molecules of distinct origin: SLs are produced through plant metabolism, whereas KARs arise from the combustion of plant carbohydrates and are thought to mimic an endogenous ligand, whose chemical identity remains unknown. Despite these differences, they share structural similarities and partially overlapping signalling pathways, which are central to understanding their crosstalk as well as their distinct *vs* shared effects. These similarities extend from structural features and sensing mechanisms to downstream signal transduction. Accordingly, SL and KAR pathways are not entirely independent; rather, they converge, interact, and mutually regulate one another. However, advancements as well as the interpretation of the existing literature are constrained by two unresolved issues. First, the effects of KARs are rightly tested against the effects of loss-of-function mutations of their receptor KAI2; but this does not allow discriminating against the effects of its endogenous, so far uncharacterized ligand. Second, the synthetic compounds most routinely used as analogues of SLs lack receptor specificity, with *rac*-GR24 activating both D14 (the proper SL receptor) and KAI2. This is a confounding factor that is often not considered when attributing phenotypes to either pathway. Drawing on current research, this paper reviews similarities and differences between the two pathways, with particular emphasis on their cooperative and regulatory roles in shoot, leaf and root development. It also reviews their functions and especially interactions under abiotic stresses such as drought, salinity, low-light conditions, nutrient limitation, and suboptimal temperatures, systematically examining the level of confidence and criticalities for each dataset. Finally, it discusses whether and how natural or synthetic KAR-related molecules may be used to enhance crop resilience under adverse environmental conditions.

## 1 Introduction

Signalling by hormones and hormone-like molecules is central to plant development and acclimation to environmental stresses (Yang et al., 2015; Wani et al., 2016; Huang et al., 2018). Within this broad category, evidence is mounting that two structurally related yet functionally distinct families of small molecules, strigolactones (SLs) and karrikins (KARs), may crosstalk with each other at various levels.

SLs are a class of phytohormones derived from carotenoids, that were first discovered in root exudates as inducers of seed germination in parasitic plants (Cook et al., 1966) and later shown to play also an important regulatory role in plant growth and development (Gomez-Roldan et al., 2008; Umehara et al., 2008; Waters et al., 2017). Accumulating evidence indicates also that SLs contribute significantly to plant resilience to abiotic stress. These advances are covered by recent reviews (Cardinale et al., 2018; Trasoletti et al., 2022; Kapoor et al., 2024), to which the reader is directed for detailed analyses beyond the overview we will provide here.

KARs are signalling molecules derived from the pyrolysis of plant carbohydrates. As such, they can be found in the smoke produced by burning plants and were first identified as germination promoters for fire-succession species (Flematti et al., 2004; Nelson et al., 2012). Despite their exogenous origin, KARs can also regulate plant growth, development, and responses to abiotic stress (Wang et al., 2018; Carbonnel et al., 2020a; Shah et al., 2021; Hamon‐Josse et al., 2022; Kim et al., 2022; Xu et al., 2022; Abdelrahman et al., 2023; Liu et al., 2023). Interestingly, both KARs and SLs contain a common bioactiphore, are perceived by cognate receptors and share some downstream signalling components, as detailed below. These similarities have prompted increasing interest in potential competitive, additive or synergistic interactions between KAR and SL signalling.

This review is informed by three main ideas. First, while previous reviews report SL functions or KAR effects without looking specifically at their integration, we re-examine the existing literature in light of the possibility that their signalling paths form a dynamically interconnected network converging on shared core components while maintaining distinct regulatory outputs, enabling plants to fine-tune developmental and stress responses in a context-dependent manner. Second, we evaluate the published evidence for crosstalk or inconclusive/confounding results according to the analogues and mutants used in each study, and tabulate these findings explicitly. Third, we consider how a low-cost source of KARs, such as smoke water—typically produced by dissolving smoke from plant material into water, could help to translate experimental outcomes into crop management options. In support of this perspective, we first briefly compare the structural and signalling similarities that underlie pathway convergence, referring the readers to previous reviews for more detailed aspects of biosynthesis and broader hormonal interactions (Ou et al., 2026). We then examine how SL and KAR signalling paths interact across key developmental processes and under major abiotic stresses, focussing on their shared signalling modules, functional differentiation, and context-dependent crosstalk.

As a methodological note, our analysis adopts a broad operational definition of “crosstalk”, encompassing shared signalling machinery, direct pathway cross-regulation, biosynthetic regulation, convergence on a common hormonal or transcriptional target, parallel or additive activity without demonstrated molecular interaction, and proposed but untested interactions. The reported observations will be classified into such sub-categories, as explicitly as possible.

## 2 Structural similarities and shared signalling components

The biosynthesis of SLs starts from all-*trans*-β-carotene (**Figure 1A**), which the key enzyme DWARF27 (D27) converts to 9-*cis*-β-carotene (Alder et al., 2012). Subsequently, two cleavage reactions occur. The first is catalysed by the key enzyme carotenoid cleavage dioxygenase 7 (CCD7), also called MORE AXILLARY GROWTH 3 (MAX3) in *Arabidopsis thaliana* (Arabidopsis), which cleaves it to produce 9-*cis*-β-apo-10′-carotenal and β-ionone. The second, catalysed by CAROTENOID CLEAVAGE DIOXYGENASE 8 (CCD8), also called MORE AXILLARY GROWTH 4 (MAX4), converts the carotenal to carlactone, a key precursor of SLs. In Arabidopsis, SLs are synthesized from carlactone via a series of enzymatic steps involving MORE AXILLARY GROWTH 1 (MAX1) and other downstream enzymes. For a comprehensive overview of the biosynthesis of SLs, see recent reviews (Trasoletti et al., 2022; Mashiguchi and Yamaguchi, 2026; Niu et al., 2026; Ou et al., 2026).

**Figure 1:**
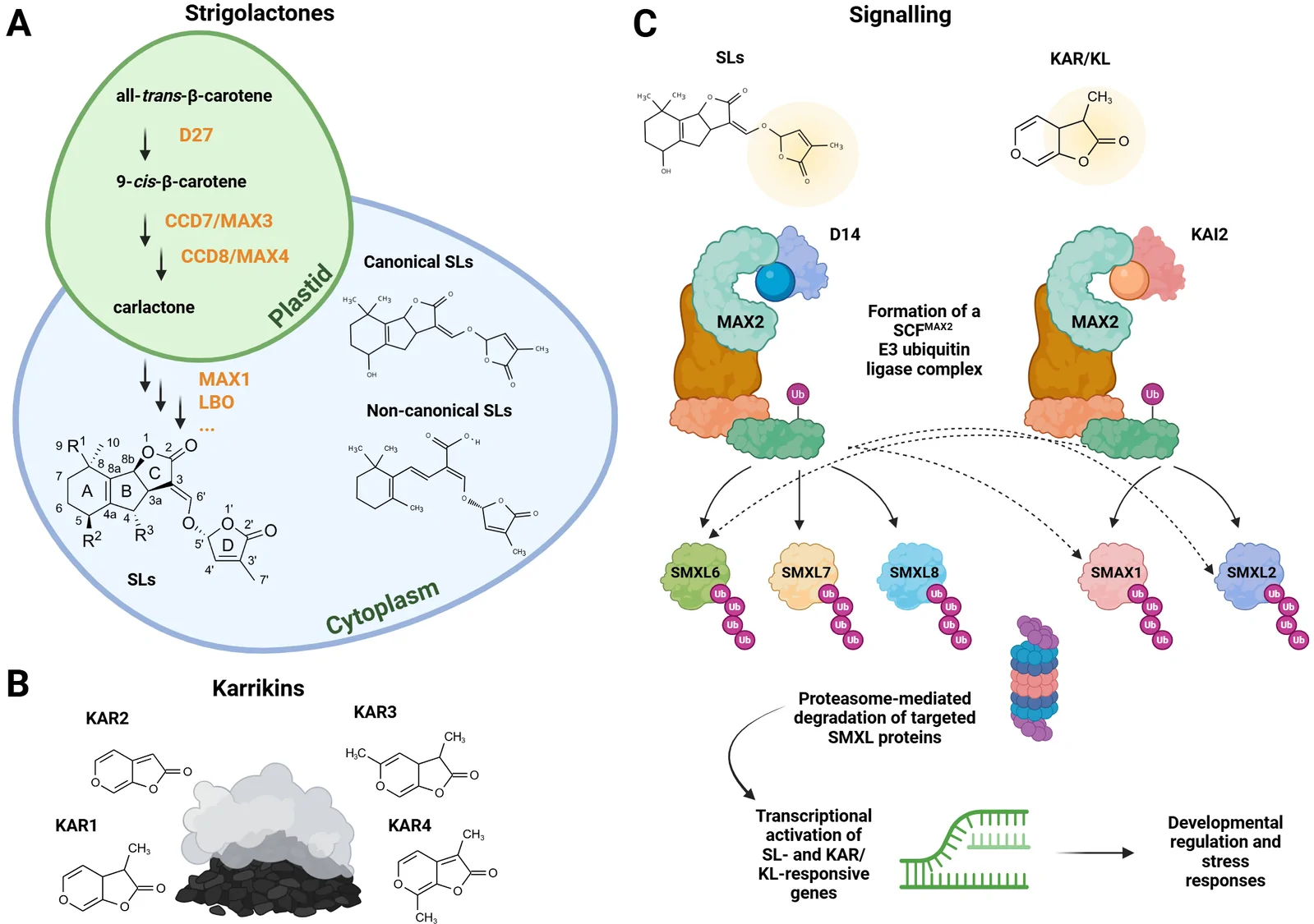
Chemical structure, biosynthetic origin and signalling mechanisms of karrikins and strigolactones. **(A)** Strigolactone (SL) biosynthesis. In the plastid, all-*trans*-β-carotene is converted to 9-*cis*-β-carotene by DWARF27 (D27), and then to carlactone by CAROTENOID CLEAVAGE DIOXYGENASE 7 (CCD7, also called MAX3) and CCD8 (MAX4). In the cytoplasm, carlactone is further modified by MORE AXILLARY GROWTH 1 (MAX1), LATERAL BRANCHING OXIDOREDUCTASE (LBO) and other enzymes to form canonical and non-canonical SLs. The core SL structure is a tricyclic ABC core connected through an enol-ether bridge to a butenolide D-ring; carbon positions and the variable groups R1–R3 are indicated. The pathway downstream of carlactone can vary considerably across species. **(B)** Karrikins (KARs; KAR1–KAR4) are butenolide compounds found in the smoke produced by burning vegetation. Their structure consists of a bicyclic system, with six-membered pyran ring fused to a five-membered butenolide ring. **(C)** Perception and signalling. Despite their differences, SLs and KARs share a common bioactiphore, highlighted in yellow. SLs are perceived by the receptor DWARF14 (D14), and KARs, or the unidentified endogenous ligand KL, by KARRIKIN INSENSITIVE 2 (KAI2). Each ligand-bound receptor associates with MORE AXILLARY GROWTH 2 (MAX2) to form an SKP1–Cullin–F-box (SCF)^MAX2^ E3 ubiquitin ligase complex. This promotes polyubiquitination and proteasome-mediated degradation of the target repressors, mainly SUPPRESSOR OF MAX2 1 (SMAX1) and SMAX1-LIKE 2 (SMXL2) for the KAI2 branch, and SMXL6, SMXL7 and SMXL8 for the D14 branch. Solid arrows show the main targets of each branch; dashed arrows indicate that some targets can be recognised by both branches under specific conditions. Loss of these repressors de-represses downstream genes, which control developmental regulation and stress responses. Ub, ubiquitin.

The structural differences among SLs are mainly due to modifications of the ABC rings, such as hydroxylation and methylation, and to the closure of the ABC rings, which is incomplete in the subgroup of SLs called “non-canonical” (as opposed to “canonical”, in which it is complete; **Figure 1A**). However, the D-ring structure, which is essential for their biological activity, is relatively conserved and is considered the key domain for their function (bioactiphore), together with the enol-ether bridge to the ABC rings (Wang and Bouwmeester, 2018). Compared with SLs, the structure of KARs is simpler: it consists in fact of a six-membered pyran ring fused with a five-membered butenolide ring, forming a bicyclic system. Typical representatives, such as KAR_1_ (3-methyl-2H-furo[2,3-c]pyran-2-one), are depicted in **Figure 1B**. A relatively detailed description of these structural features can be found in the literature (Waters et al., 2013; Waters and Nelson, 2023).

Thus, the most striking structural similarity between the two classes of molecules is that both contain a butenolide ring (Wang et al., 2022). This shared chemical motif serves as the structural basis for their recognition by receptors of the same superfamily (**Figure 1C**). In fact, the structural similarity between SLs and KARs is mirrored by their respective receptors, DWARF14 (D14) and KARRIKIN INSENSITIVE 2 (KAI2). Both these proteins belong to the α/β-hydrolase superfamily; they are similar in both sequence and especially three-dimensional structure, with KAI2 generally believed to be ancestral to D14 (Waters et al., 2012). The same superfamily also includes D14-LIKE2 (DLK2), a further paralogue that retains an intact catalytic triad but has lost the surface residues required for MAX2 binding; it is inferred to catabolise butenolide ligands without transducing a signal, as discussed in §5 (Li et al., 2026). The existence of an endogenous ligand for KAI2, provisionally named KL for ‘KAI2 ligand’, is strongly supported by genetic and phenotypic analyses of *kai2* mutants (Waters et al., 2012). Complementary structure–activity relationship studies involving systematic testing of diverse butenolide scaffolds, various substituents, D-ring–modified analogues, and SL-like molecules, demonstrate that KAI2 preferentially recognizes compounds containing a butenolide ring, as expected (Kushihara et al., 2025). Thus, the elusive endogenous KL likely harbours a butenolide moiety itself, although, pending its identification, direct chemical evidence remains lacking (Conn and Nelson, 2016; Kushihara et al., 2025).

Upon SL perception and hydrolysis, D14 undergoes a conformational change that initiates transduction by making the activated D14 interact with the F-box protein MORE AXILLARY GROWTH 2 (MAX2) to form the E3 ubiquitin ligase complex SKP1–Cullin–F-box MAX2 (SCF^MAX2^) (**Figure 1C**). This complex recruits the ubiquitination machinery to proteins in the broad SUPPRESSOR OF MAX2 1 (SMAX1)-LIKE (SMXL) family, leading to their degradation and to the relief of SMXL-mediated transcriptional repression (Chang et al., 2025). KAI2 shares a similar downstream signalling architecture involving the formation of a complex with SCF^MAX2^ and the subsequent degradation of transcriptional regulators that belong to the same SMXL family. New evidence indicates that KARs may require metabolic conversion prior to activating signalling, in order to mimic the endogenous KL (Dubois, 2022; Sepulveda et al., 2022; Waters and Nelson, 2023; Moe‐Lange and Shabek, 2025).

In Arabidopsis, phylogenetic analysis suggests that members of the broad SMXL family likely originated from the ancestral *SMAX1* gene through ancient gene duplication and functional divergence events (Moturu et al., 2018). Among them, SMAX1 and SMXL2 are the primary targets for SCF^MAX2^ downstream of KAI2 activation (Stanga et al., 2013; Soundappan et al., 2015), while the D14 pathway mainly targets SMXL6/7/8 (Soundappan et al., 2015; Wang et al., 2015). It is worth noting however, as discussed later in more detail, that SMAX1 and SMXL2/6 can be targeted by both signalling cascades under specific conditions, supporting the notion that the two pathways are not insulated from each other, even downstream of MAX2 (Wang et al., 2020).

Finally, two issues pertaining ligand structures and shared receptors currently limit what can be concluded from the existing body of work on the topic. First, because natural SLs are difficult to extract at scale due to their low abundance and relatively unstable structure, the synthetic analogue GR24 is widely used in research. Structure-activity studies established that the GR24^5DS^ and GR24^4DO^ stereoisomers activate the D14 signalling pathway, while GR24*^ent^*^-5DS^ (the unnatural stereoisomer) preferentially activates the KAI2 signalling pathway (Scaffidi et al., 2014; Wang et al., 2020). Thus, the racemic mixture *rac*-GR24, which contains GR24^5DS^ and GR24*^ent^*^-5DS^, can activate both KAI2- and D14-dependent signalling pathways, and results obtained with *rac*-GR24 should be interpreted with caution, especially with regard to crosstalk between the two pathways (Flematti et al., 2016; Wang et al., 2020). This criticality is systematically accounted for in **Supplementary Table S1**, which includes all case studies we critically reviewed along with the availability of genetic data in support of pathway attribution. As an additional confounding factor, as mentioned above, the endogenous ligand KL has not been chemically identified yet, along with its biosynthetic pathway. Thus, we lack both KL-specific biosynthetic mutants and pure KL for treatments. For this reason, and because the effects of loss-of-function mutations on *KAI2* will inevitably entail loss of both KAR and KL signalling, the hypothesis that KAR and KL effects are completely overlapping in any relevant context cannot be conclusively proven or disproven yet at the experimental level.

## 3 Cooperation and crosstalk in growth and development

Once either KAI2 or D14 are activated, they can regulate various stages of plant growth and development. Notably, the abovementioned convergence on shared signalling components, such as MAX2 and SMXL proteins, suggests a potential basis for coordinated regulation. However, despite this shared signalling framework, they frequently exert distinct or even contrasting effects on specific developmental processes, indicating context-dependent interactions that integrate both cooperation and functional divergence. The aspects of growth and development in which such interactions are observed are surveyed here to follow, from which it appears that D14 and KAI2 attain co-ordinate regulation through receptor-specific outputs. In addition, signalling crosstalk and mutual interference arise from partially overlapping ligand recognition and shared components, and evidence is emerging for interaction also at the biosynthetic level, as described below.

### 3.1 Shoot morphology

In higher plants, SLs are well established as negative regulators of shoot branching and tillering (**Figure 2A**; Gomez-Roldan et al., 2008; Umehara et al., 2008). However, the exact structure of the endogenous branching-inhibitory signal remains unresolved, and increasing evidence suggests that canonical SLs may not represent the main *in planta* branching signal (Chen et al., 2023; Dun et al., 2023; Wang et al., 2023, 2024). Instead, non-canonical SL derivatives downstream of carlactone have been proposed as potential candidates, although their precise identity and mode of action remain to be clarified (Dun et al., 2023; Wang et al., 2024). Evidence for a direct and conserved role of the KL signalling pathway in shoot branching remains preliminary, and its mechanistic basis is less well understood than that of SLs. In rice, for instance, disruption of the putative KAI2 orthologue DWARF14-LIKE (D14L), which mediates responses to GR24*^ent^*^-5DS^ rather than to the SL-like GR24^5DS^ (Kagiyama et al., 2013; Zheng et al., 2020), has been reported to influence plant architecture, with loss-of-function mutants showing fewer tillers and reduced shoot growth alongside enhanced root development and an increased root-to-shoot ratio (**Figure 2A**; Salem et al., 2025). These observations suggest that D14L signalling does contribute to shoot development, potentially because it affects whole-plant resource allocation. Available evidence further indicates that this influence on tillering is context-dependent, being most evident under phosphate (Pi)-limiting conditions (Salem et al., 2025). However, beyond parallel regulation of the same physiological process (though to opposite outcome: SL-related mutants show increased tillering in rice), pharmacological or genetic proof of crosstalk between the pathways in the context of branching/tillering is currently missing.

**Figure 2:**
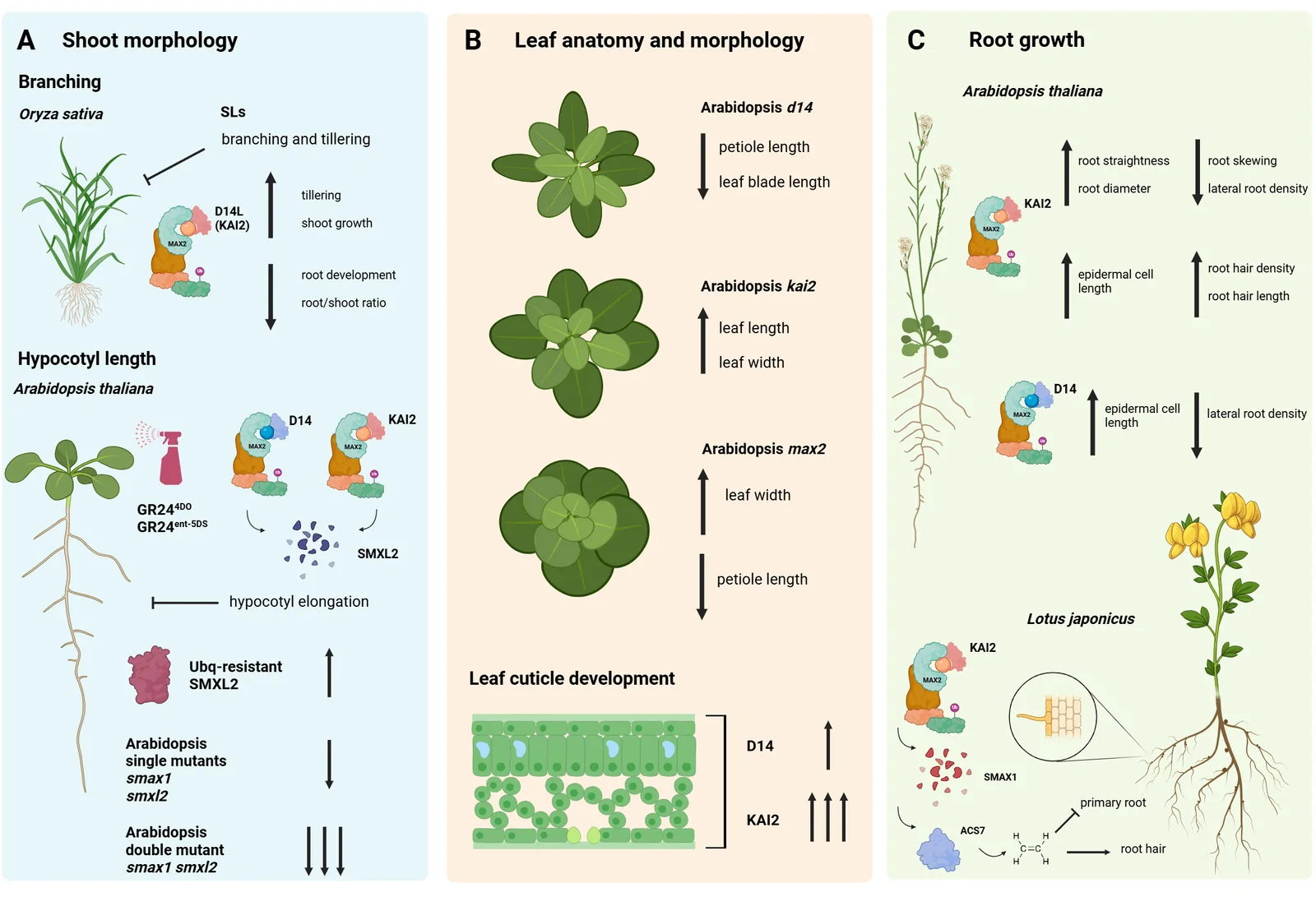
Roles of strigolactone and karrikin signalling in plant growth and development. **(A)** Shoot morphology. In rice (*Oryza sativa*), strigolactones (SLs) suppress branching and tillering, whereas DWARF14-LIKE (D14L) signalling increases tillering and shoot growth and decreases root development and the root-to-shoot ratio. In *Arabidopsis thaliana*, the GR24 stereoisomers GR24^4DO^ (perceived by D14) and GR24*^ent^*^-5DS^ (perceived by KARRIKIN INSENSITIVE 2 - KAI2) both inhibit hypocotyl elongation. Hypocotyl length is increased by a ubiquitination-resistant form of SUPPRESSOR OF MAX2 1 (SMAX1)-LIKE 2 (SMXL2), reduced in the *smax1* and *smxl2* single mutants, and further reduced in the *smax1 smxl2* double mutant. **(B)** Leaf anatomy and morphology. The *d14* mutant shows reduced petiole and leaf blade length; the *kai2* mutant has increased leaf length and width; the *max2* mutant of the F-box protein-encoding *MORE AXILLARY GROWTH 2* shows increased leaf width and reduced petiole length. D14 and KAI2 promote leaf cuticle development, with KAI2 exerting the strongest effect. **(C)** Root growth. In Arabidopsis, KAI2 increases root straightness, root diameter, epidermal cell length, root hair density and root hair length, and decreases root skewing and lateral root density; D14 increases epidermal cell length and decreases lateral root density. In *Lotus japonicus*, KAI2–MAX2 signalling promotes SMAX1 degradation, relieving its suppression of ethylene biosynthesis; the resulting 1-AMINOCYCLOPROPANE-1-CARBOXYLATE SYNTHASE 7 (ACS7)-dependent ethylene production inhibits primary root growth and promotes root hair formation. Arrows indicate an increase (↑) or decrease (↓); barred lines indicate inhibition.

When it comes to early shoot development instead, particularly photomorphogenesis and the inhibition of hypocotyl elongation under continuous red light, there is clear pharmacological and genetic evidence in Arabidopsis that both D14- and KAI2-dependent pathways participate through partially overlapping downstream components (**Figure 2A**). At the transcriptional level, GR24^4DO^ induces the expression of *SMXL2* in a D14-dependent manner, whereas the stereoisomer GR24*^ent-^*^5DS^ induces the expression of *SMAX1* and *SMXL2* via KAI2. Although the two ligands act through different receptors and induce largely different sets of *SMXL* genes, *SMXL2* is induced by both, providing a first indication that the two pathways may share SMXL2 as a common downstream node (Wang et al., 2020). Whether this shared induction is functionally relevant to hypocotyl elongation, however, is resolved at the protein level. Co-immunoprecipitation assays confirmed that GR24^4DO^ enhances the interaction between D14 and SMXL2, while GR24*^ent^*^-5DS^ promotes SMXL2 association with KAI2. Genetic evidence also backs receptor-specific activation by either ligand: GR24^4DO^ -induced SMXL2 degradation was blocked in *d14-1*, and in *kai2–2* its GR24*^ent^*^-5DS^-triggered degradation was strongly impaired. Thus, both pharmacological and genetic evidence strongly support the convergence of distinct signalling pathways on SMXL2 degradation. However, it must be noted that receptor preference is not absolute in this setup. Some SMXL2 degradation still occurred in the single mutants and disappeared completely only in the *d14–1 kai2–2* double mutant, which likely means that each ligand can also use the other receptor to a small extent (Wang et al., 2020).

The functional significance of these findings is further supported by phenotypic analyses. Under continuous red light, both GR24^4DO^ and GR24*^ent^*^-5DS^ enhanced hypocotyl shortening in wild-type Arabidopsis seedlings, and the two compounds had additive effects. Seedlings overexpressing a degradation-resistant form of SMXL2 (35S:SMXL2Δ-GFP, which lacks the RGKT motif required for ubiquitination upon MAX2 interaction) developed longer hypocotyls and became insensitive to both stereoisomers, while the corresponding degradation-resistant version of SMXL6 had no such effect on photomorphogenesis. Degradation of SMXL2 is therefore required for the hypocotyl response to red light, and represents the node at which D14- and KAI2-dependent signalling converge for this trait (Wang et al., 2020).

However, SMXL2 is not the only relevant target in the family as far as hypocotyl development of Arabidopsis: SMAX1 and SMXL2 can be functionally redundant, and the two can compensate for each other (Stanga et al., 2016). Genetic analyses support this: the hypocotyls of the *smax1* or *smxl2* single mutants are only slightly shorter than the wild type, whereas in the *smax1 smxl2* double mutant, they are much shorter. Because GR24*^ent^*^-5DS^ also induces an interaction between KAI2 and SMAX1, SMAX1 is likely degraded in parallel with SMXL2 and acts in a similar way, although its degradation has not yet been directly demonstrated in this context (Wang et al., 2020).

### 3.2 Leaf anatomy and morphology

In terms of leaf morphology, the phenotypic effects of D14- and KAI2-dependent signalling show a certain degree of complementarity (**Figure 2B**). In SL biosynthetic mutants and in *d14*, both petiole and leaf blade lengths are reduced, whereas in *kai2* mutants, both the length and width of the leaves are increased. The *max2* mutant shares the general rosette morphology of *d14*, including shorter petioles, but has a rounder and wider blade than *d14*, consistent with a hybrid of the two single-mutant phenotypes (Soundappan et al., 2015). At the anatomical level, both pathways promote leaf cuticle development in Arabidopsis (**Figure 2B**), but KAI2 signalling seems to have a more profound effect that could be a driver for the efficiency of acclimation responses to water stress, as detailed in §4.2 (Li et al., 2017). In any case, leaf anatomy and morphology seem to be a sound case in which, in spite of the shared transducers, the two pathways manage to attain distinct outcomes.

### 3.3 Root growth

Both D14- and KAI2-dependent signalling are involved in the regulation of root growth and development, as they both inhibit lateral root formation and promote root hair elongation (**Figure 2C**). The attribution of these phenotypes to one pathway or the other, however, has a tangled history that is worth making explicit. Many of the earliest reports of SL effects on root system architecture—reduced lateral root density together with increased root hair density and length—were based either on the application of the synthetic analogue *rac*-GR24 or on the analysis of *max2* mutants (Kapulnik et al., 2011; Koltai, 2011; Ruyter-Spira et al., 2011). Both tools are now known to be insufficiently specific to distinguish between the two pathways. As noted above, *rac*-GR24 activates both signalling pathways simultaneously (Scaffidi et al., 2014); likewise, MAX2 is shared, so *max2* phenotypes cannot be ascribed to SL signalling alone. When the contributions of the two pathways were subsequently dissected with stereochemically pure GR24 isomers and pathway-specific mutant backgrounds, a substantial fraction of the root and root-hair phenotypes originally attributed to SLs proved to depend on KAI2 signalling. More in detail, KAI2 alone can regulate root hair density and length, as well as root skewing, straightness, and diameter, whereas both KAI2 and D14 together control lateral root density and epidermal cell length (Villaécija-Aguilar et al., 2019). Thus, this is an instance of genetically and pharmacologically supported overall pathway specificity, with some indication of functional overlap only for specific features. The untested but likely explanation for this very partial overlap is that components of the signalling machinery, probably by combinatorial associations among themselves and with other transducers, manage to direct part of the informational flow from shared to pathway-specific outcomes.

Support for a conserved role of KAI2-dependent signalling in root hair development also comes from *Lotus japonicus* (**Figure 2C**). Here, KAI2-dependent signalling leads to degradation of the SMAX1 orthologue. SMAX1 normally suppresses ethylene biosynthesis: its removal raises ethylene levels by de-repressing the biosynthetic gene *1-AMINOCYCLOPROPANE-1-CARBOXYLATE SYNTHASE 7* (*ACS7*), thus promoting root hair growth (Carbonnel et al., 2020a). Notably, this mechanism for root hair elongation appears to be conserved, but its activation by KAI2 is context- and species-specific. In *L. japonicus*, the *smax1* root phenotype persists despite changes in nutrient availability (Carbonnel et al., 2020a). In Arabidopsis, by contrast, KAI2- and MAX2-dependent root hair elongation happens mainly as a response to Pi starvation, which raises their expression, although loss of SMAX1 and SMXL2 lengthens root hairs constitutively even at high Pi (Villaécija-Aguilar et al., 2022). These findings do not exclude important D14-dependent functions in root biology but suggest that KAI2-dependent signalling accounts for a larger proportion of root developmental phenotypes than previously recognised.

## 4 Signalling and crosstalk under environmental stress

In addition to the cooperative interactions observed in the regulation of plant growth and development, recent studies indicate multiple points of interaction between SLs and KARs in responses to abiotic stress as explained below. At the moment, broader and more convincing claims of improved stress acclimation seem to be available for SLs (Trasoletti et al., 2022) than KARs (Fang et al., 2023). However, this imbalance may be only apparent, as many of such SL-related claims are based not so much on the phenotypes under stress of pathway-specific mutants, or the use of SL-specific analogues, but on the effects of treatments with *rac*-GR24. The following sections therefore distinguish, wherever possible, between evidence obtained from pathway-specific genetic and pharmacological analyses *vs* inconclusive results under this respect (an overview and systematic comparison of the published findings examined in this review can be found in **Supplementary Table S1**).

### 4.1 Nutrient starvation

Both D14- and KAI2-dependent signalling pathways contribute to plant acclimation to nutrient limitation, particularly under phosphorus (P) and nitrogen (N) deficiency (**Figure 3A**). It has been long known that SL biosynthesis and exudation increase when P or N availability is low (Yoneyama et al., 2007, 2012). In rice, low P increases SL biosynthesis through the module comprising PHOSPHATE STARVATION RESPONSE 2 (PHR2) and NODULATION SIGNALING PATHWAY 1/2 (NSP1/NSP2) (Yuan et al., 2023; Hong et al., 2026). The PHR transcription factors activate both SL biosynthetic genes directly and *NSP* genes – which then further reinforce SL biosynthesis.

**Figure 3:**
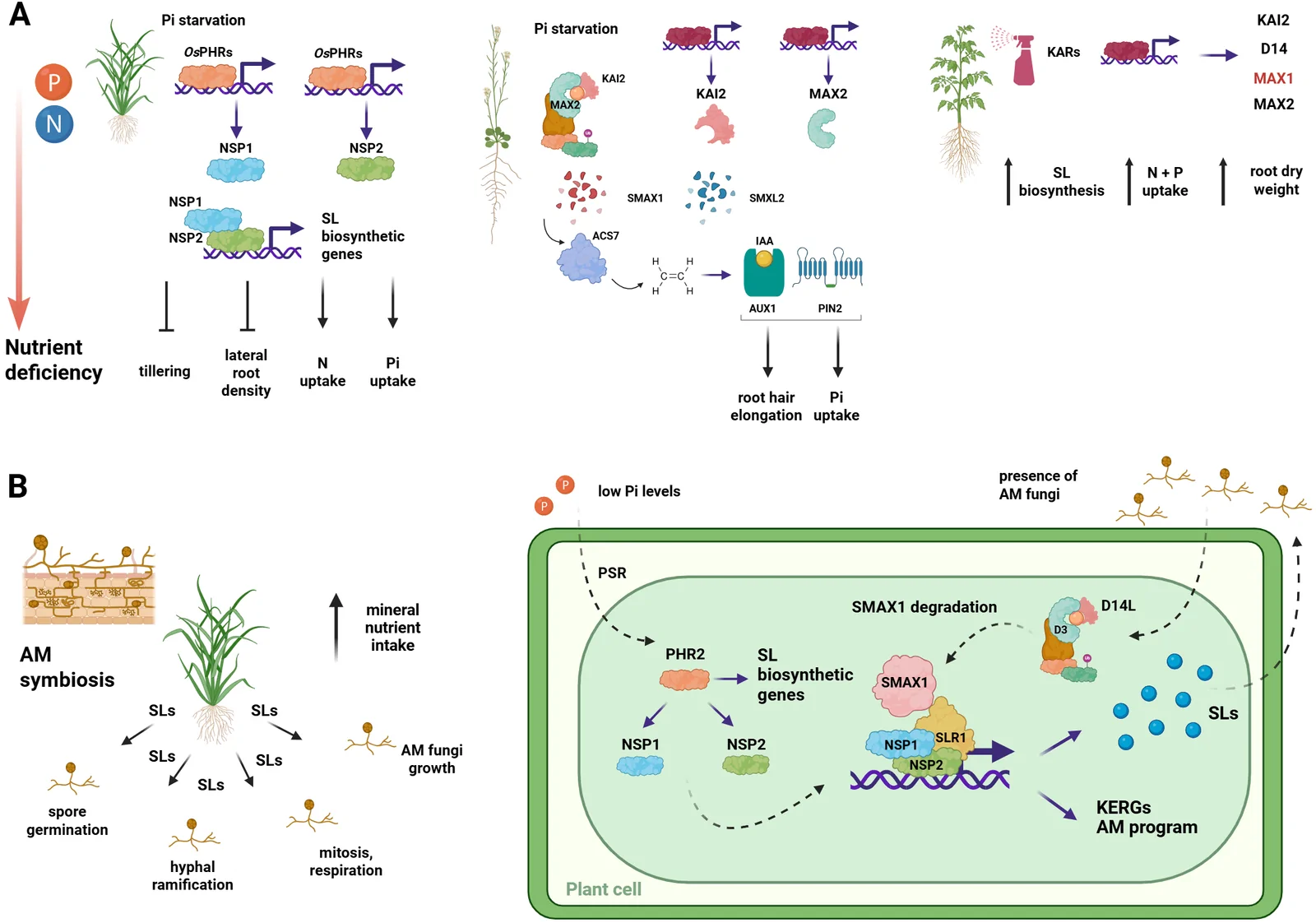
Strigolactone and karrikin signalling in nutrient-starvation responses and arbuscular mycorrhizal symbiosis. **(A)** Nutrient-deficiency responses. The vertical red arrow on the left denotes decreasing nitrogen (N) and phosphorus (P) availability, *i.e.* increasing nutrient deficiency. In rice under P starvation, PHOSPHATE STARVATION RESPONSE (PHR) transcription factors induce NODULATION SIGNALING PATHWAY 1 (NSP1) and NSP2, which together activate strigolactone (SL) biosynthetic genes; this suppresses tillering and lateral root formation while promoting N and P uptake. In *Arabidopsis thaliana* under P starvation, the KARRIKIN INSENSITIVE 2 (KAI2)–MORE AXILLARY GROWTH 2 (MAX2) complex promotes degradation of SUPPRESSOR OF MAX2 1 (SMAX1) and SMAX1-LIKE 2 (SMXL2); the downstream 1-AMINOCYCLOPROPANE-1-CARBOXYLATE SYNTHASE 7 (ACS7)-dependent ethylene production and auxin (indole-3-acetic acid, IAA) transport through AUXIN TRANSPORTER PROTEIN 1 (AUX1) and PIN-FORMED 2 (PIN2) drive root hair elongation and P uptake. In tomato, application of karrikins (KARs) induces the signalling genes *KAI2*, *DWARF14* (*D14*) and *MAX2*, together with the SL biosynthetic gene *MAX1* (shown in red); this increases SL biosynthesis, N and P uptake and root dry weight. **(B)** Arbuscular mycorrhizal (AM) symbiosis. SLs released from roots promote spore germination, hyphal ramification, fungal mitosis and respiration, and AM fungal growth, thereby enhancing the host’s mineral nutrient intake. At the cellular level, low phosphate (Pi) triggers the Pi starvation response (PSR): PHR2 induces SL biosynthetic genes and the NSP1 and NSP2 transcription factors. In the presence of AM fungi in rice, the D14-LIKE (D14L)–DWARF3 (D3) receptor complex promotes SMAX1 degradation; relief of SMAX1 repression allows the NSP1–NSP2–SLENDER RICE 1 (SLR1) complex to activate SL biosynthesis and the KAR Early Responsive Genes (KERGs)/AM transcriptional programme. Except when otherwise specified by the sprayer icon, all data presented in the figure is derived from genetic evidence, as summarised in **Supplementary Table S1**.

In turn, D14- and KAI2-dependent signalling shapes the nutritional stress response (**Figure 3A**). Work with SL-deficient and SL-signalling mutants in rice shows that SL biosynthesis and downstream signalling through DWARF3 (D3) are required for root and tiller plasticity under phosphate and nitrate limitation (Umehara et al., 2010; Sun et al., 2014). In Arabidopsis, KAI2 promotes root hair elongation at low Pi by controlling the auxin carriers AUXIN TRANSPORTER PROTEIN 1 (AUX1) and PIN-FORMED 2 (PIN2) (Villaécija-Aguilar et al., 2022) through the degradation of SMAX1 and the subsequent induction of ethylene-mediated auxin transport, in a system that is equivalent to that described for *Lotus japonicus* in §3.3 (Carbonnel et al., 2020a). In tomato, KARs increase N and P uptake, root dry weight and the root-to-shoot ratio in the absence of nutrient depletion. Importantly, these effects likely occur through a KAR-controlled SL and abscisic acid (ABA) signalling network that was shown to orchestrate also temperature-stress responses, with the SL biosynthesis gene *MAX1* working downstream of KAI2 (see also §4.3) (Liu et al., 2023). Thus, KARs do not seem to act alone in these species, but to regulate nutrient acquisition partly by switching on SL signalling, something that has been more clearly demonstrated in the case of root symbioses.

Indeed, the two pathways mutually influence each other in a way that can modulate the odds and intensity of root colonisation by arbuscular mycorrhizal (AM) fungi (**Figure 3B**); this, in turn, will affect nutrient acquisition and plant fitness under low availability. Indeed, SLs are also signals that plants exude into the rhizosphere to recruit AM fungi before contact, by stimulating spore germination, hyphal branching, mitosis, respiration and growth in the fungal partner, thus increasing the chances of contact (Akiyama et al., 2005; Besserer et al., 2006, 2008). In line with this, rice mutants in SL biosynthesis show reduced or delayed colonisation, even though the fungal structures that do form look normal (Gutjahr et al., 2012; Kobae et al., 2018). So, a D14L-dependent signal that modulates SL production also changes the plant’s ability to attract and support the AM fungus (Gutjahr et al., 2015; Hull et al., 2021; Meng et al., 2022; Das et al., 2025).

As it happens, the D14L-D3 receptor complex is required for AM formation in rice (Gutjahr et al., 2015), and its proteolytic target SMAX1 represses not only the AM programme itself but also SL biosynthesis. So when D14L signalling degrades SMAX1, it does two things at once: it raises SL production, which attracts the fungus, and it switches on AM-related genes, which prepares the root for colonisation (Choi et al., 2020; Hull et al., 2021). Hong et al. (2026) provide a comprehensive analysis of how nutrient status, in particular low Pi levels, feeds into this system (**Figure 3B**): an array of genes designated as KAR-signalling-regulated early response genes (KERGs; involved in SL biosynthesis as well as in other aspects of AM symbiosis) are controlled by the transcriptional complex comprising NSP1–NSP2–SLENDER RICE 1 (SLR1, the rice DELLA protein), and this in turn is repressed by SMAX1. D14L-mediated signalling, activated by AM fungi, lifts SMAX1 repression at the same time as the transcription factor PHR2 promotes the expression of both NSP proteins—reinforcing KERG activation—and of SL biosynthetic genes directly (Hong et al., 2026).

SMAX1 therefore works in rice as a gatekeeper where stress signals and D14L-dependent signalling together set the level of SL production and AM colonisation. This wiring between D14L/KAI2-dependent signalling and SL biosynthesis via SMAX1 seems to be specific to AM host plants. It is absent in the non-host Arabidopsis (Mashiguchi et al., 2023), while it is confirmed in *L. japonicus*, where it was additionally proven that ethylene stabilises SMAX1 thus inhibiting both AM symbiosis and SL biosynthesis (Das et al., 2025). Such findings are consistent with the hypothesis that this regulatory connection evolved in association with the establishment of the AM symbiosis, and are an example of direct pathway cross-regulation at the biosynthetic level.

The effects of smoke water under P deficiency deserve some dedicated comments as well. Smoke water, which contains KARs among its bioactive ingredients, has been shown to promote root elongation in rice without markedly increasing total plant P levels (Pinit et al., 2023). Whether pure KARs affect nutrient levels in rice has not been tested; in tomato, by contrast, KARs increase N and P uptake (§4.1; Liu et al., 2023). This suggests that its growth-promoting effect is not simply due to enhanced nutrient acquisition. Instead, evidence points to a role in modulating stress responses, including activation of detoxification-related enzymes such as glutathione-S-transferases and reduced ROS accumulation. In addition, changes in the expression of photosynthesis-related and SL-biosynthetic genes have been reported upon smoke water treatment, indicating a broader reprogramming of stress and developmental responses (Pinit et al., 2023). Further work at the cellular level indicates that smoke water enhances both meristem activity and cell elongation, thereby contributing to increased root length under P limitation. Interestingly, this is accompanied by a suppression of root hair elongation and a reduction in P uptake rates; but treated plants show higher P utilization efficiency, producing more biomass per P unit and exhibiting altered internal P distribution, with relatively greater allocation to roots (Ariyakulkiat et al., 2026). The possibility that smoke water might improve P acquisition not only by reshaping the root but also by promoting mycorrhizal colonisation and SL metabolism, however, still needs direct testing.

In summary, available evidence suggests that KARs, via KAI2 (and, possibly, smoke water), positively affect nutrient use through crosstalk with SLs, including along the SL–AM axis.

### 4.2 Osmotic stress and salinity

Both D14- and KAI2-dependent signalling are important for effective acclimation responses to drought (**Figure 4A**), and this appears to happen, in part, via deep integration in the metabolism and perception processes of ABA, a key hormone for the execution of such responses. For example, the shared MAX2 transducer engages in extensive crosstalk with ABA signalling. In Arabidopsis, loss-of-function *max2* mutants exhibit pronounced hypersensitivity to drought and decreased sensitivity to exogenous ABA, whereas SL biosynthetic mutants (*max3* and *max4*) present weaker phenotypes; this led initially to discard the possibility that SLs indeed affect drought acclimation (Bu et al., 2014). Nonetheless, currently available evidence amply demonstrates that SLs do exert a positive regulatory role in plant responses to drought (Ha et al., 2014; Visentin et al., 2016, 2020, 2025; Li et al., 2020; Korwin Krukowski et al., 2022; Daszkowska-Golec et al., 2023; Shu et al., 2023; Cao et al., 2024; Moosavi et al., 2025). Likewise, increasing evidence supports a positive role for KAI2 in drought acclimation. Rather than acting redundantly, D14- and KAI2-dependent signalling pathways appear to contribute to drought acclimation through partially distinct mechanisms (Li et al., 2017): these include convergent changes to ABA metabolism and sensitivity, cuticle formation and stomatal closure for the reduction of water loss, the accumulation of antioxidant compounds and stress memory mechanisms that influence recovery and the response to new stress events.

**Figure 4:**
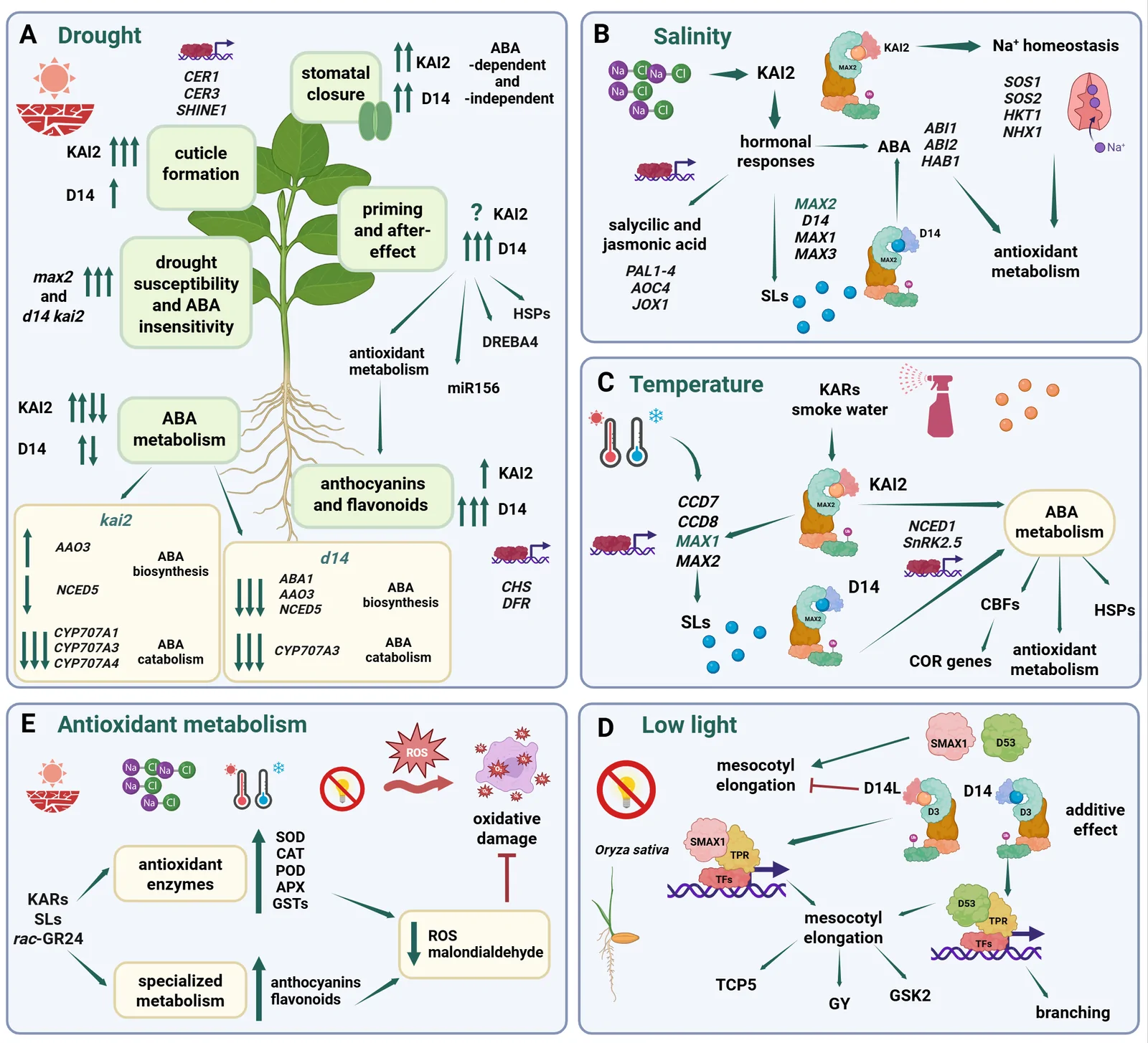
Strigolactone and karrikin signalling in plant responses to abiotic stress. **(A)** Drought. Both DWARF14 (D14) and KARRIKIN INSENSITIVE 2 (KAI2) signalling contribute to drought acclimation, largely through crosstalk with abscisic acid (ABA). KAI2 contributes more strongly to cuticle formation, upregulating cuticle biogenesis genes such as *ECERIFERUM1* (*CER1*), *ECERIFERUM3* (*CER3*) and *SHINE1* (*SHN1*), whereas D14 contributes more strongly to anthocyanin and flavonoid accumulation, e.g. by upregulating *CHALCONE SYNTHASE* (*CHS*) and *DIHYDROFLAVONOL 4-REDUCTASE* (*DFR*). Loss of both pathways (in *max2* and *d14 kai2* mutants) increases drought susceptibility and ABA insensitivity. The two pathways also reshape ABA metabolism: the *kai2* background shows strong downregulation of ABA catabolic genes *CYP707A* (ABA 8′-hydroxylases) and, to a lesser extent, dysregulation of the ABA biosynthetic gene *AAO3* (*ABSCISIC ALDEHYDE OXIDASE 3*). The *d14* background shows a more strongly reduced expression of ABA biosynthetic genes *ABA1* and *AAO3*, and shares with *kai2* the downregulation of ABA catabolic genes of the *CYP707A* family. D14 signalling, but not KAI2 as far as currently known (indicated by “?”), is linked to drought priming and after-effects through the induction of heat shock proteins (HSPs) and other targets, including microRNA156 (miR156). Evidence obtained mostly with *rac*-GR24 treatments also indicates an ABA-independent mechanism for stomatal closure under drought. **(B)** Salinity. KAI2 contributes to salt tolerance and is required to induce SL biosynthesis genes *MORE AXILLARY GROWTH 1* and *3* (*MAX1*, *MAX3)* and signalling genes *MAX2*, *D14*. It also affects ABA-responsive genes such as *ABA-INSENSITIVE 1* and *2* (*ABI1*, *ABI2*) or *HYPERSENSITIVE TO ABA 1* (*HAB1*), directly and through SLs. In addition, KAI2 contributes to osmotic stress acclimation by promoting ion homeostasis through the expression of genes such as *SALT OVERLY SENSITIVE 1* and *2* (*SOS1*, *SOS2*), *HIGH-AFFINITY K^+^ TRANSPORTER 1* (*HKT1*) and *Na^+^/H^+^ EXCHANGER 1* (*NHX1*), which, together with ABA, indirectly supports redox homeostasis. **(C)** Temperature. Under both high and low temperature, SL biosynthesis is promoted by the upregulation of biosynthetic genes such as *CAROTENOID CLEAVAGE DIOXYGENASE 7* and *8* (*CCD7*, *CCD8*) and *MAX1*, and of the signalling component *MAX2*. KARs/smoke water perceived by KAI2 also regulate SL levels by promoting *MAX1* expression. Both D14- and KAI2-mediated signalling upregulate ABA biosynthesis and sensitivity through transcriptional activation of *NCED1* and *SNF1-RELATED PROTEIN KINASE 2.5* (*SnRK2.5*). ABA induces cold/heat-responsive genes (heat shock proteins and cold-responsive genes), as well as antioxidant metabolism, to improve tolerance. **(D)** Low light. In rice grown in the dark, the D14-LIKE (D14L)–D3 and D14–D3 complexes act additively to control mesocotyl elongation. D14L–D3 targets SUPPRESSOR OF MAX2 1 (SMAX1) for degradation, while D14–D3 targets D53; both repressors target genes involved in mesocotyl elongation, such as *TEOSINTE BRANCHED1/CYCLOIDEA/PROLIFERATING CELL FACTOR 5* (*TCP5*), *GOLIATH 1* (*GY1*) and *GLYCOGEN SYNTHASE KINASE 2* (*GSK2*). **(E)** Antioxidant metabolism. Abiotic stresses such as drought, salinity, temperature and low light all affect cell homeostasis by promoting the formation of reactive oxygen species (ROS) that cause oxidative damage. Evidence shows that *rac*-GR24 treatments can raise the activity of antioxidant enzymes (superoxide dismutase, SOD; catalase, CAT; peroxidase, POD; ascorbate peroxidase, APX; glutathione S-transferases, GSTs) and of specialised metabolism (production of anthocyanins, flavonoids), lowering ROS and malondialdehyde (MDA), protecting cell membranes and reducing oxidative damage. Arrows indicate an increase (↑) or decrease (↓); barred lines indicate inhibition; “?” marks a relationship that remains unresolved. When the portrayed effects are attributed mainly (but not necessarily exclusively) to *rac-*GR24, rather than to a specific receptor/pathway, this is specified and relationships are indicated by red arrows. The sprayer icon indicates result obtained following treatments with KARs or smoke water. All other data presented in the figure is derived from genetic evidence, as summarised in **Supplementary Table S1**.

Thus, the stronger drought sensitivity phenotype observed in *max2* than *max3* or *max4* mutants may be due to the double loss of D14 and KAI2, that help the plant cope with drought via only partially overlapping mechanisms (Li et al., 2020). Indeed, a systematic comparison in Arabidopsis using *d14*, *kai2*, and the *d14 kai2* double mutant indicated that both receptors contribute positively to drought tolerance, and the double mutant was more sensitive than either single mutant (Li et al., 2020). Anthocyanin accumulation and expression of their biosynthetic genes *CHALCONE SYNTHASE (CHS)* and *DIHYDROFLAVONOL 4-REDUCTASE (DFR)* were reduced in *d14*, and to a lesser extent in *kai2*, whereas cuticle biogenesis genes *ECERIFERUM 1 (CER1), ECERIFERUM 3 (CER3)* and *SHINE 1 (SHN1)* were more strongly affected in *kai2* than *d14* (Li et al., 2020). Importantly, both pathways also modulate ABA metabolism, albeit through partly distinct targets: the biosynthetic genes *ABA DEFICIENT 1 (ABA1)* and *ABSCISIC ALDEHYDE OXIDASE 3 (AAO3)* responded mainly—though not exclusively—to loss of D14, whereas the catabolic *ABA 8′-hydroxylase (CYP707A)* genes, and *CYP707A3* in particular, were affected more strongly in *kai2*. Transcriptome comparison under irrigated and dehydrated conditions also showed that the two mutants alter largely distinct sets of ABA-related genes (Li et al., 2020). These are examples of convergence on common hormonal and transcriptional targets via a parallel and additive activity without demonstrated molecular interaction, besides of course at the level of MAX2.

Overall, the D14 and KAI2 functional modules show both differences and complementarity in regulating ABA biosynthesis and accumulation: *D14* seems to have a comparatively greater effect on ABA biosynthesis, while *KAI2* may mostly affect ABA catabolism (Li et al., 2020). It must be noted here that whether SL signalling raises or lowers ABA content depends on the species, the mutant and the growth conditions. For example, *kai2*, *d14* and *max2* Arabidopsis plants accumulate more ABA than the wild type, especially but not exclusively under prolonged drought, whereas barley (*Hordeum vulgare*) mutants show no change, SL-depleted tomato accumulates less ABA and SL-deficient *L. japonicus* shows no clear change unless other stresses occur simultaneously (Bu et al., 2014; Liu et al., 2015; Visentin et al., 2016; Li et al., 2020). These contrasting observations highlight that the relationship between D14 or KAI2 signalling and ABA accumulation is highly context dependent, and varies with the species, developmental stage and environmental conditions. GR24, on the other hand, has been reported to induce not only higher ABA sensitivity but also a metabolite increase in several cases (Russo et al., 2023). Thus, it is somewhat surprising that stomatal closure driven by *rac*-GR24 was proven to be independent of ABA perception in Arabidopsis (Lv et al., 2018). However, the D14-selective enantiomers, which were since demonstrated to induce stomatal closure in other species (Visentin et al., 2020), were not directly tested in the same system, so we cannot exclude that stomatal closure in ABA-insensitive mutants was due to KAI2-dependent signalling instead (even though, the effects of *rac*-GR24 were lost in the *d14* mutant) (Lv et al., 2018). Indeed, whether KARs or the KAI2-selective GR24 enantiomers can transiently and directly close stomata, and whether they would require ABA sensing to do so, is currently unclear.

More generally, recent findings indicate that the relationship between the two pathways under osmotic stress is not strictly linear. Under osmotic stress conditions, in fact, D14 has also been shown to target SMAX1 for degradation, revealing a degree of signalling plasticity that extends beyond the canonical separation between D14–SMXL6/7/8 and KAI2–SMAX1/SMXL2 modules. This is an instance of direct pathway cross-regulation, at least under the tested conditions (Li et al., 2022).

As a further emerging facet, SLs have been shown to influence the physiological and transcriptional memory of drought, *i.e.* the priming and/or lingering effect (also known as after-effect) that a previous stressful event has on the responses to a subsequent episode, and/or in the immediate aftermath of a single episode. Of note, SL signalling is linked to proper induction and priming of genes encoding heat shock proteins and factors, which are key not only for thermotolerance but also for efficient acclimation to a range of environmental stresses (Visentin et al., 2025). One of the mechanisms through which both endogenous and externally supplied SLs act during drought is to induce miR156, whose overexpression in turn amplifies the after-effect and promotes sensitivity to ABA (Visentin et al., 2020). The potential involvement of KAI2-mediated signalling in this phenomenon, however, remains unknown (Visentin et al., 2020, 2025; Korwin Krukowski et al., 2022; Mostofa et al., 2023; Salem et al., 2025), opening interesting questions as to whether and how KAI2-dependent signalling participates in stress memory formation and maintenance at the molecular and physiological level, including possible interactions with the SL pathway in this context.

A similar pattern of reduced resistance in both *kai2* and SL-related mutants is observed under salinity stress (Ha et al., 2014; Shah et al., 2020; Trasoletti et al., 2022; Fang et al., 2023; Agliassa et al., 2025; Wang et al., 2025). This is accompanied by a similar crosstalk pattern observed under other environmental stress conditions (**Figure 4B**). For example, in Arabidopsis, KAI2 is not only needed to upregulate the expression of *MAX2* and *D14* but also to robustly induce transcription of *MAX1* and *MAX3* when the plant is exposed to high salt, indicating that KAI2-dependent signalling integrates with SL biosynthesis to jointly enhance salt tolerance. Because *MAX2* is the shared hub of both pathways, and its transcript level is markedly reduced in *kai2* mutants, SL signalling is correspondingly affected under high salinity in the absence of KAI2 function (Mostofa et al., 2023). Moreover, KAI2 also acts in salinity tolerance by regulating ABA-responsive genes: *ABA-Insensitive 1* and *2*(*ABI1,2*), along with *Hypersensitive to ABA 1* (*HAB1*) are less expressed due to KAI2 inactivity, and there are indications that other hormonal pathways may be affected. In the same study, it was also observed how KAI2 plays a role in regulating ion homeostasis in response to salt stress: the maintenance of a stable K^+^/Na^+^ balance is one of the major mechanisms of osmotic adjustment, which is regulated, among others, by the high-affinity K^+^ transporter (HKT), the Na^+^/H^+^ antiporter (NHX) and SALT OVERLY SENSITIVE (SOS) proteins. In *kai2* mutants, the activation of *SOS*, *HKT* and *NHX* genes upon salt stress was strongly reduced or delayed. Ion homeostasis is itself a fundamental component of the cell’s ability to control oxidative damage, further highlighting how KAI2-dependent signalling (and possibly its interweaving with D14 signalling) is central to the regulation of antioxidative capacity and multi-hormone responses (Mostofa et al., 2023). There are no clear indications that SLs may directly regulate the same target genes under salinity stress, as the sole published datasets on which the connection is openly drawn rely on the use of *max2* mutants or *rac*-GR24 (Mehrabi et al., 2024; Sousa et al., 2026). Both pathways however converge on the regulation of antioxidant metabolism, as detailed in §4.5.

### 4.3 Temperature stress

Both D14- and KAI2-dependent signalling take part in plant responses to suboptimal temperatures (**Figure 4C**). On the SL side, for example, both high and low temperatures induce the expression of the biosynthetic genes *CCD7*, *CCD8* and *MAX1* in tomato, as well as of *MAX2*. Consistently, silencing them reduces both heat and cold tolerance, possibly due to altered ABA homeostasis and sensitivity (Chi et al., 2021). ABA in turn regulates the antioxidant metabolism (Zhang et al., 2006; Hu et al., 2010), as well as transcription of *HEAT SHOCK PROTEIN 70* (*HSP70*) and of *C-REPEAT BINDING FACTOR 1* (*CBF1*) (Knight et al., 2004; Hu et al., 2010). The latter transcription factor is then known to promote the expression of *COLD-REGULATED (COR)* genes that mediate acclimation to non-freezing low temperatures by enhancing the synthesis of cryoprotective metabolites (Jaglo-Ottosen et al., 1998).

The contribution of KAI2-dependent signalling has been studied mainly under cold. In Arabidopsis for example, *kai2* mutants are very sensitive to cold or freezing temperatures, whereas overexpression of a *KAI2* orthologue from *Sapium sebiferum* increases tolerance by improving the expression of *CBF* genes and redox homeostasis (Shah et al., 2021). In tomato, KARs are perceived through KAI2 to promote cold tolerance, with the SL biosynthesis gene *MAX1* being activated and acting downstream of KAR. This KAI2–D14 connection via MAX1 improves cold acclimation, again, by regulating ABA biosynthesis and sensitivity, namely through the *9-*cis*-epoxycarotenoid dioxygenase 1* (*NCED1*) and *SNF1-RELATED PROTEIN KINASE 2.5* (*SnRK2.5*) components, respectively. Interestingly, KARs seem able to regulate *SnRK2.5* expression both through SL biosynthesis and independently of *MAX1* expression, directly impacting on ABA responses (Liu et al., 2023). This is therefore an example of both distinct and overlapping contributions to stress tolerance by the two pathways, attained by parallel targeting of shared genes and hormonal pathways, as well as cross-pathway biosynthetic regulation.

### 4.4 Low-light stress

Both pathways impinge on skotomorphogenesis, inhibiting it (**Figure 4D**). In rice under dark conditions, the mesocotyl length of the *d14l* loss-of-function mutant was similar to that of the *d14* mutant—both were longer than that of the wild type but shorter than that of the *d3* mutant. The mesocotyl length of the *d14 d14l* double mutant was comparable to that of *d3* (Zheng et al., 2020; Hu et al., 2014). Transcriptomic analysis of dark-grown seedlings revealed highly similar expression changes in *d3* and *d14 d14l* compared with the wild type, which strongly supports the concept that the signalling pathways initiated by both receptors converge on D3 to slow mesocotyl elongation of dark-grown rice seedlings (Zheng et al., 2020).

Subsequent yeast two-hybrid and pull-down assays demonstrated physical interaction between the rice D14L and SMAX1 proteins. KAR-induced degradation of SMAX1 required D14L and D3 but not D14, as expected (Zheng et al., 2020). The study of overexpressing and knockout lines further confirmed that the abundance of SMAX1 modulates genes such as *TEOSINTE BRANCHED1/CYCLOIDEA/PROLIFERATING CELL FACTOR 5* (*TCP5*), the putative *GOLIATH 1* (*GY1*) orthologue, and *GLYCOGEN SYNTHASE KINASE 2* (*GSK2*), which are associated with mesocotyl length. Moreover, excess SMAX1 suppresses the expression of *D14L*, *D14L2*, and *D14L3*, as does the loss of *D14L* itself, which lowers the expression of its paralogues *D14L2* and *D14L3*. Together, these findings indicate that the D14 and D14L pathways act additively and in parallel in the regulation of gene expression, jointly controlling downstream genes to coordinate developmental processes under dark conditions in rice (Zheng et al., 2020). Notably, however, the two pathways also cross-regulate each other: loss of *D14* suppresses *D14L* expression, and the D14L-dependent pathway in turn influences SL signalling, since *DWARF53 (D53)* transcripts are reduced in *d14l* and *SMAX1m-OE* plants (overexpressing a stabilized, gain-of-function version of *SMAX1*).

Consistent with such points of convergence, knockout of *SMAX1* completely rescued the elongated mesocotyl of the *d3* mutant, indicating that the accumulation of SMAX1 is the main driver of this phenotype in rice. Knockout of *SMAX1* leads to a similarly shortened mesocotyl phenotype in SL biosynthesis and perception mutants, such as *d10* (impaired in the *MAX4* orthologue function) and *d14* (Choi et al., 2020; Zheng et al., 2020). To add to the intricacy, the expression of *SMAX1* is positively regulated by the D14-D3-D53 axis but negatively regulated by its own protein product; thus, while it is downregulated in *d14* and in plants overexpressing a stabilized form of *D53* (*D53m-OE*), it is upregulated in the *smax1 d14* double mutant. *D53* transcription is also feedback-regulated by D14- and D14L-dependent signalling: its expression is reduced in *d14* and in *D53m-OE* lines, but also in the *d14l* and *SMAX1m-OE* genotypes; and it is increased in *smax1* mutants. Collectively, these results indicate that the D14–D3–D53 and D14L–D3–SMAX1 signalling complexes act interdependently to maintain the expression levels of *D53* and *SMAX1* in dark-grown rice seedlings, in a clear instance of cross-pathway transcriptional regulation of signalling components (Zheng et al., 2020).

### 4.5 Redox homeostasis as a convergence point across stresses

All stresses considered above converge on oxidative damage caused by reactive oxygen species (ROS) and lipid peroxidation (**Figure 4E**). Redox homeostasis therefore represents a mechanism shared across these conditions rather than a stress-specific response, and is treated here comparatively; the individual findings are given in sections 4.1 to 4.4, and the topic has been reviewed separately for both signals (Trasoletti et al., 2022; Fang et al., 2023).

The enzymatic component is the best documented, although the available evidence rests largely on *rac*-GR24. Under drought, *rac*-GR24 applied alone or together with salicylic acid increases superoxide dismutase, peroxidase, catalase and ascorbate peroxidase activity and membrane stability in winter wheat (Sedaghat et al., 2017). Under salinity, comparable increases in antioxidant enzyme activity and reductions in malondialdehyde have been reported in oilseed rape and wheat (Ma et al., 2017; Mehrabi et al., 2024). Under low light, *rac*-GR24 similarly raised antioxidant enzyme activity and lowered hydrogen peroxide and malondialdehyde in cucumber seedlings (Zhou et al., 2022). SL-deficient and SL-insensitive mutants are correspondingly impaired in antioxidant metabolism (Bu et al., 2014; Wang et al., 2019; Marzec et al., 2020; Agliassa et al., 2025; Visentin et al., 2025; Wani et al., 2026). A comparable pattern is reported for KAR treatment and inferred from *kai2* mutants (Li et al., 2017; Shah et al., 2021; Mostofa et al., 2023). Non-enzymatic defences follow the same pattern: flavonoid and anthocyanin accumulation is reduced in SL-deficient mutants and in *kai2* and *max2* under water deficit (Ha et al., 2014; Li et al., 2017), and proline is less abundant in SL-depleted tomato plants exposed to high salt (Agliassa et al., 2025).

Two qualifications apply across these studies. Most (although, not all) SL-related experiments used *rac*-GR24, so the enzymatic responses cannot be convincingly assigned to SL signalling alone; and *max2* phenotypes shared by both pathways are often behind the evidence. The apparent convergence of D14 and KAI2 effects on redox homeostasis may therefore be partly methodological and awaits more testing with stereopure analogues in receptor-specific backgrounds. At present, however, this rather appears to be an area of parallel or additive activity converging on shared targets, without demonstrated molecular interaction at the signalling or biosynthetic level.

## 5 From stress acclimation to agronomic potential: open questions and unresolved issues

Several open questions remain, here ordered by the extent to which resolving each would inform the others.

First and foremost, a knowledge asymmetry persists at the ligand-level: whereas the endogenous biosynthetic routes and molecular structures of SLs are relatively well defined, the endogenous KL has yet to be identified, and its chemical structure, stress-induced dynamics, and spatiotemporal distribution throughout development remain unresolved. To complicate the matter further, in some experiments, KAR_1_ or KAR_2_ weakly or slowly activate the KAI2 pathway, while GR24*^ent^*^-5DS^ can trigger a stronger activation. This difficulty in precisely and reliably activating the KAR pathway remains an obstacle to the study of the effects of pulse treatments (Wang et al., 2020). Until a candidate ligand is isolated, KAI2 function can only be inferred from loss-of-function phenotypes, and the relationship between KAR responses and endogenous signalling cannot be established. Activity-guided fractionation in *kai2* backgrounds and comparative metabolomics of *kai2* and wild-type tissue are the least-resistance paths to address this; but whether KL is hydrolysed by KAI2 or binds without turnover is itself unresolved and would alter how such screens are designed. Additionally, recent studies indicate that KARs may require metabolic conversion to generate bioactive signals perceived by KAI2, and that the resulting bioactive metabolite may itself be, or mimic, the endogenous KL. Two negative feedback mechanisms that restrict KAR/KL signalling have now been described in Arabidopsis. The first involves KAR UPREGULATED F-BOX 1 (KUF1): mutations in this gene alter sensitivity to KAR₁, but not to KAR₂ or GR24ent-5DS, which led to the proposal that KUF1 acts upstream of KAI2–SCFMAX2 and negatively regulates both the biosynthesis of endogenous KL and the conversion of KAR₁ into an active ligand of KAI2 (Sepulveda et al., 2022). The second involves DLK2, an ancient paralogue of KAI2 and D14 that retains an intact catalytic triad but has lost the surface residues required for MAX2 binding (Li et al., 2026). Loss of DLK2 alone has little consequence, yet it synthetically enhances the constitutive KAR/KL responses of kuf1; both KUF1 and DLK2 stabilise a SMAX1 reporter without affecting an SMXL7 reporter, which indicates that this feedback is specific to the KAI2 branch rather than to SCFMAX2-dependent signalling in general. DLK2 hydrolyses a desmethyl butenolide substrate thought to resemble KL, but not its methyl counterpart, and remains functional when excluded from the nucleus; it is therefore inferred to catabolise KAI2 ligands rather than to transduce a signal (Li et al., 2026). Of direct relevance to the identification of KL, the kuf1 dlk2 double mutant, defective in both feedback arms, has been proposed as an enriched source of the ligand.

The second issue concerns ligand selectivity across receptors and species. KAI2 clades have expanded independently in several lineages, and the resulting paralogues may act redundantly or differ in ligand preference. Findings from Arabidopsis, which carries a single *KAI2*, therefore transfer poorly to species in which the relevant paralogue has not been identified yet (Bythell-Douglas et al., 2017); for most crops, it is not known which paralogue(s) perceives KL, which perceives KARs, and whether they are the same protein. Cross-species complementation of Arabidopsis *kai2*, together with panels of stereopure analogues, could start addressing this with existing tools. However, the repertoire of ligands perceived by D14 and KAI2 appears broader and more dynamic than previously thought. For instance, some KAI2 paralogues from *Petunia* × *hybrida* appear to very specifically perceive volatile terpenoids that are crucial for floral organ development (Stirling et al., 2024). Also, evidence from pea (*Pisum sativum*) demonstrates that certain KAI2 paralogues (*e.g.*, *Ps*KAI2B) have broadened stereoselectivity and are capable of perceiving and hydrolysing SL-type stereoisomers such as GR24^5DS^, which are generally considered D14-specific (Guercio et al., 2022). This suggests that ligand specificity between D14- and KAI2-dependent signalling pathways may not be strictly conserved across species, further complicating the interpretation of crosstalk when synthetic analogues are used (Guercio et al., 2022; Trasoletti et al., 2022). Even structurally unrelated, microbial molecules can be sensed by D14 (Chen et al., 2026). The picture is made even more complex by the existence of endogenous ligands that do not simply activate these receptors: the apocarotenoid zaxinone binds the active site of D14—and, more weakly, of KAI2—but acts as a long-lasting, non-hydrolysable antagonist that blocks the D14–MAX2 interaction rather than promoting it (Moreno et al., 2025).

A further open question concerns the strong context dependence of KAI2- and D14-mediated responses, that can act in opposite directions on a single process depending on the environment. For example, studies on the *kai2* mutant in Arabidopsis show that KAI2 activity promotes seed germination under optimal conditions but inhibits it under abiotic stress such as osmotic stress, salinity or elevated temperature, thereby preventing germination under conditions unfavourable to seedling establishment (Wang et al., 2018). Beyond such environmental switching, the function of KAI2 also differs between species and between organs, which suggests considerable evolutionary and tissue-level plasticity in how the pathway is deployed (Carbonnel et al., 2020b). On the SL side, as an example, exogenous SLs are known to promote stomatal closure transiently in unstressed plants (Visentin et al., 2016, 2020; Lv et al., 2018; Zhang et al., 2018) and more persistently in abruptly dehydrated plants, when stress avoidance mechanisms are the only ones that can be reasonably deployed (Visentin et al., 2016, 2020). However, if dehydration is more progressive, there is a window of opportunity for SLs to actually promote mitigation strategies—such as better redox and protein homeostasis—leading to better stress tolerance, that ultimately correlates with more open stomata over time (Sedaghat et al., 2017; Trasoletti et al., 2022). The dynamics of stress onset are indeed known to heavily influence acclimation strategies (Morabito et al., 2022, 2025; Trasoletti et al., 2022).

It also remains to be established how inputs from distinct receptors that are forced through the shared MAX2 bottleneck still manage to translate into receptor-specific transcriptional outputs. How is appropriate target selection achieved at the MAX2–SMXL hub, where the same F-box protein directs degradation of (mostly) SMAX1 and SMXL2 following KAI2 activation, and of (mostly) SMXL6/7/8 following D14 activation? Whether specificity resides in direct receptor–target contact, in receptor–MAX2 conformation, or in tissue-specific expression has not been distinguished experimentally yet. We are also just beginning to appreciate the different facets of hormonal crosstalk triggered by KAI2 and D14 signalling separately: although both pathways participate in feedback circuits with ABA, evidence is appearing about effects on well-established hormonal networks. Although the precise transcriptional control nodes and the molecular interfaces that mediate these cross-pathway connections are still poorly defined (Chi et al., 2021; Liu et al., 2022, 2023), this is an area where we could expect indirect mutual influence between the KAI2 and D14 pathways.

As mentioned throughout, a non-insignificant part of the achieved progress remains inferential, and rests on compounds and mutant backgrounds that cannot distinguish the two pathways. This ambiguity is especially common for research with applicative purposes, where the immediate goal of improving plant performances under realistic conditions is normally met with the use of *rac*-GR24. While the mixed signals in its commercial preparations may be seen as an advantage from a practical standpoint (after all, both pathways may contribute to the final outcome, and the mixture is less costly and more readily available than pure enantiomers), the lack of awareness regarding this specific point often misleads result interpretation and affects the prospects and limitations for the use of SL analogues, KARs and smoke water in agriculture. Although the chemical synthesis of KARs is not as challenging as the one of natural SLs, their use is not (yet) economically viable for the agrochemical market either—especially for crops on which mark-ups are low. However, as mentioned earlier, smoke water contains a mixture of bioactive compounds, among which KARs are considered key contributors (Flematti et al., 2004; Van Staden et al., 2004), and it could be seen as a cheap, easily available alternative to pure KARs as crop management tools.

At present, smoke water shows potential for the adoption in agricultural practices, that have traditionally employed smoke exposure to a variety of purposes (Kulkarni et al., 2011). Previous studies have demonstrated that treatments can significantly improve seedling emergence and vigour in crops such as tomato, okra (*Abelmoschus esculentus*), and maize (*Zea mays*) (Kulkarni et al., 2007; Elsadek and Yousef, 2019), and that this can occur also under environmental stress (Jain and Van Staden, 2006; Płażek et al., 2018). In addition, foliar application of smoke water has been reported to enhance physiological performance and increase essential oil yield in *Mentha arvensis* (Singh et al., 2023). Thus, smoke water, owing to its low cost and broad applicability, represents a promising candidate for practical agricultural use. However, as an inherently complex mixture, its mode of action remains insufficiently resolved. Its efficacy can certainly be affected by several factors, among which plant material and production conditions; batch-to-batch differences in composition; dose-dependent inhibitory as well as stimulatory effects; stability, storage, formulation, and application method; differences between laboratory, greenhouse, and field performance; crop-, genotype-, and developmental-stage specificity. Thus, it is not surprising that its effects may be hard to reproduce and do not always completely overlap with those of purified KARs (Soós et al., 2010; Zhou et al., 2014), which highlights the need for standardised chemical characterisation and application methods.

Last but not least, any stress-relief tool that we wish to add to our crop management solutions should be carefully tested under field conditions. Almost all available data derive from single-factor experiments under controlled or semi-controlled conditions, whereas realistic conditions impose stresses concurrently, and the current evidence suggests that interactions may not be predictable from single-stress data. This is a prerequisite for any agronomic use of SL- or KAR-related compounds, smoke water included.

## 6 Conclusions

Even though the scientific community has barely started addressing the point, the current body of evidence indicates that KAI2- and D14-dependent signalling can crosstalk with each other at several levels, forming a coordinated but non-redundant regulatory network, and one that is highly context dependent. The two pathways share core signalling components, can regulate each other, and act synergistically and/or complementarily during both development and stress responses. They happen to operate in parallel under some conditions and hierarchically under others, with KAI2 signalling often acting upstream of SL biosynthesis in rice, *Lotus japonicus* and tomato. Obviously, more work is needed to disentangle the contribution of each pathway by the use of specific signalling mutants and synthetic analogues, as a clear prerequisite for the informed, precise and effective use of such molecules in field applications.

## Glossary

AAO3: ABSCISIC ALDEHYDE OXIDASE 3
ABA: Abscisic acid
ABA1: ABA DEFICIENT 1
ABI: ABA INSENSITIVE
ACS7: 1-AMINOCYCLOPROPANE-1-CARBOXYLATE SYNTHASE 7
AM: Arbuscular mycorrhiza/arbuscular mycorrhizal
APX: ascorbate peroxidase
AUX1: AUXIN TRANSPORTER PROTEIN 1
CAT: catalase
CBF: C-repeat binding factor
CCD: CAROTENOID CLEAVAGE DIOXYGENASE
CER: ECERIFERUM
CHS: CHALCONE SYNTHASE
COR: Cold REGULATED Genes
CYP707A: ABA 8′-hydroxylase
D3: DWARF3
D10: DWARF10
D14: DWARF14
D14L: DWARF14-LIKE
D27: DWARF27
D53: DWARF53
DELLA: DELLA-domain protein
DFR: DIHYDROFLAVONOL 4-REDUCTASE
DLK2: DWARF14-LIKE2
GR24: Synthetic strigolactone analogue
GR24^4DO^: 4-deoxy-GR24
GR24^5DS^: 5-GR24
GR24*^ent^*^ - 5DS^: *ent* - 5-GR24
GSK2: Glycogen synthase kinase 2
GST: Glutathione S-transferase
GY1: GOLIATH 1
HSP: Heat shock protein
HAB1: HYPERSENSITIVE TO ABA 1
HKT: HIGH-AFFINITY K⁺
KERG: KAR EARLY RESPONSIVE GENE
IAA: Indole-3-acetic acid (auxin)
KAI2: KAR INSENSITIVE 2
KAR: Karrikin
KL: KAI2 ligand
KUF1: KAR UPREGULATED F-BOX 1
LBO: LATERAL BRANCHING OXIDOREDUCTASE
MAX: MORE AXILLARY GROWTH
MDA: Malondialdehyde
miR156: microRNA156
N: Nitrogen
NCED1: NINE-*CIS*-EPOXYCAROTENOID DIOXYGENASE 1
NHX: Na⁺/H⁺
NSP: NODULATION SIGNALING PATHWAY
P: Phosphorus
Pi: Inorganic phosphate
PIN2: PIN-FORMED 2
POD: peroxidase
PSR: phosphate starvation response
ROS: reactive oxygen species
SCF^MAX2^: SKP1–Cullin–F-box MAX2 complex
SHN1/SHINE1: SHINE1, an AP2/ERF transcription factor
SL: strigolactone
SLR1: SLENDER RICE 1
SMAX1: SUPPRESSOR OF MAX2 1
SMXL: SMAX 1-LIKE
SnRK2.5: SNF1-RELATED PROTEIN KINASE 2.5
SOD: superoxide dismutase
SOS: SALT OVERLY SENSITIVE
TCP5: TEOSINTE BRANCHED1/CYCLOIDEA/PCF 5
Ub: ubiquitin
