# Supplementary material for: Crosstalk between strigolactone and KAI2/KL signalling in plant growth and stress responses
Source: Front Plant Sci. 2026 Sep 21;17:1931114. doi: 10.3389/fpls.2026.1931114 (PMC13636905; doi:10.3389/fpls.2026.1931114)
Supplement: Supplementary file 1 [file DataSheet1.pdf]

Supplementary Table S1. Species, tissue and evidence class underlying the mechanisms discussed in this review, with an assessment of conservation versus divergence.

Process / output (section)	Species	Tissue, stage and conditions, main results	Biosynthetic, receptor and downstream components tested as knockout/down	Evidence class and tools, evidence of crosstalk	Conserved, species-specific or divergent	Reference
Suppression of shoot branching and tillering (§3.1)	Arabidopsis, pea, rice	Axillary buds (pea 10 d; Arabidopsis 23–48 d, spanning bolting) and tiller buds (rice 2–6 weeks) de-inhibited in relevant mutants, compensated by exogenous treatments.	CCD7, CCD8, MAX1, MAX2/RMS4/D3.	**A:** biosynthetic mutants with hormone replacement. GR24* rescued *ccd7*, *ccd8* and *max1* but not *rms4*, *max2* or *d3*; (+)-strigol and (+)-5-deoxystrigol gave the same results in rice.<br>**Crosstalk:** not addressed.	Conserved in all three species. Confirmed in numerous other species since.	Gomez-Roldan et al., 2008; Umehara et al., 2008
Tillering, shoot and root growth, and grain yield under contrasting Pi (§3.1)	Rice	Stage-dependent. No difference between genotypes up to 3 weeks of age; reduced height and tillers at 6 weeks under low Pi only; under sufficient Pi, only from booting. Root architecture was assessed separately, on intact root systems grown from seed in soil-filled rhizotrons and imaged non-destructively 3–4 weeks after sowing under a single Pi supply.	D14L, reported here as OsKAI2.	**B:** two CRISPR nulls (*d14l-1*, *d14l-3*) in hydroponics and soil, each at sufficient vs low Pi. No ligand, no complementation.<br>**Crosstalk:** biosynthetic regulation. SLs drop in *d14l* root tissue and exudates, which also lose *Striga*-germinating activity, so D14L sustains SL biosynthesis (in line with Choi et al., 2020; Mashiguchi et al., 2023).	Conditional on Pi, stage, allele and environment, e.g. no tillering defect was later found in paddy-grown *d14l* with a different allele (Zheng et al., 2020).	Salem et al., 2025
Promotion of hypocotyl shortening under continuous red light (§3.1)	Arabidopsis	Hypocotyls of 4- to 5-d-old seedlings; protoplasts and *35S:SMXL2-GFP* seedlings for protein work. D14 and KAI2 both drive SMXL2 degradation, dependent on its RGKT motif; SMAX1 acts redundantly. SMXL6/7/8 do not contribute.	MAX1, MAX3, MAX4, D14, KAI2, SMAX1, SMXL2/6/7/8.	**A:** stereopure GR24 isomers on *d14*, *kai2* and *smax1 smxl2*. KAR_1 much less effective than $GR24^{ent\text{-}5DS}$, consistent with a need for metabolic activation.<br>**Crosstalk:** convergence on a common target. SMXL2 is a shared target, degraded through KAI2 and, via $GR24^{5DS}$, through D14.	Not broadly extrapolatable. SMXL2 arose from a Brassicaceae-specific duplication of SMAX1, and species outside this family carry no equivalent paralogue (Moturu et al., 2018). *max1*, *max3*, *max4* and *d14* have wt hypocotyls, so the D14 route to SMXL2 may be pharmacological, seen only under treatment with exogenous ligand.	Wang et al., 2020; Stanga et al., 2016
Petiole length and leaf blade dimensions (§3.2)	Arabidopsis, (*kai2-1* backcrossed from Ler twice)	Seventh rosette leaf of long-day plants, marked at about 4 weeks, measured at 35 d and 37 d. Whole rosettes were also photographed at 28 d in long days and at 37 d in short days. Photoperiod changes how the two branches affect leaf growth, but the short-day comparison is qualitative only Petiole length: D14 promotes elongation, SMXL6/7/8 inhibit it. The short petiole of *max2* matches *d14* and is additively restored by *smxl6*, *smxl7* and *smxl8*. KAI2 and SMAX1 have little or no effect on the petiole. Blade dimensions: both branches act here, in opposite directions. SMAX1 promotes blade	D14, KAI2, MAX2, SMAX1, SMXL2/6/7/8.	**A, B:** T-DNA alleles in higher-order combinations, mostly in *max2*; no ligand applied to leaves.<br>**Crosstalk:** parallel activity without demonstrated molecular interaction. Crosstalk was tested at the target level, negative results: KAR_1 does not destabilise SMXL7 and *d14* alone stabilises it, so KAI2 does not regulate SMXL7. The two branches converge on one organ but act through separate targets with opposite effects, and specificity is attributed to receptor–target pairing rather than to expression domains, since *SMAX1* and *SMXL6/7/8* promoters overlap in leaves.	Arabidopsis only.	Soundappan et al., 2015

Process / output (section)	Species	Tissue, stage and conditions, main results	Biosynthetic, receptor and downstream components tested as knockout/down	Evidence class and tools, evidence of crosstalk	Conserved, species-specific or divergent	Reference
		length and width, since *smax1* reduces both in *max2* and in *smxl6,7 max2*, and *kai2*, which stabilises SMAX1, has longer and wider blades. SMXL6 and SMXL7 repress blade elongation, since *smxl6,7* increases it in *max2*. Removing both target sets restores near wt leaf dimensions (*smax1 smxl6,7 max2*). In a *MAX2* background, *smxl6,7,8* alone narrows the blade and lengthens the petiole.				
Leaf cuticle development (§3.2)	Arabidopsis	Fifth rosette leaf of 4-week-old plants (TEM); whole rosette leaves at 28 d (chlorophyll leaching). KAI2 is indispensable for wt cuticles, cuticle and wax genes (*CER1*, *CER4*, *WSD1*, *MYB16*, *SHN1–3*) are downregulated in *kai2-2* while *d14-2* is indistinguishable from the wt. *d14-2* is nevertheless drought-sensitive, so the receptors drive drought resistance by partly different routes (developed further in §4.2).	D14, KAI2.	**B:** *kai2-2*, *kai2-4*, *d14-2*, *kai2-2 d14-2* and two *35S:KAI2* lines giving the reciprocal phenotype.<br>**Crosstalk:** not addressed.	Arabidopsis only.	Li et al., 2017
Root hair density and length, root skewing and diameter, lateral root density, epidermal cell length (§3.3)	Arabidopsis	Primary root epidermis of 5-day-old seedlings, 2–3 mm from the tip; lateral roots at 10 dpg. KAI2–MAX2 → SMAX1/SMXL2; SMXL2 alone accounts for root hair length and skewing, SMAX1 for diameter. Lateral root density and epidermal cell elongation affected by both branches.	MAX1, MAX3, MAX4, D14, KAI2, MAX2, SMAX1, SMXL2/6/7/8.	**A:** *max1*, *max3*, *max4*, *d14*, *kai2*, *max2*, *smax1*, *smxl2*, *smax1 smxl2*, *smxl6/7/8*, plus KAR_2 and stereopure GR24 isomers.<br>**Crosstalk:** convergence on a common target. Lateral root density requires both branches, as a sign of molecular cooperation; some indications of incomplete ligand specificity in this setup leave ligand-level crosstalk open.	Arabidopsis only, KAI2-specific under standard conditions. Supersedes earlier SL attributions based on *rac*-GR24 or *max2*. The *smxl6/7/8* skewing result replicated in only one laboratory.	Villaécija-Aguilar et al., 2019
Root hair elongation and primary root length through ethylene (§3.3, §4.1)	*Lotus japonicus*, Arabidopsis	*L. japonicus*: roots of 10-d-old seedlings. Arabidopsis: root hairs of 5-d-old seedlings at 2, 625 and 2000 µM Pi. KAI2–MAX2→ SMAX1/SMXL2→ ACS7→ ethylene. Root hair density and length require loss of both targets; diameter is suppressed by *smax1* alone; ethylene acts downstream of SMAX1/SMXL2 within the KAI2 branch.	Lotus: KAI2, MAX2, SMAX1, EIN2<br>Arabidopsis: D14, KAI2, MAX2, SMAX1, SMXL2, ACS7, EIN2.	**A:** *L. japonicus smax1*, *kai2a kai2b*, *max2*, *ein2a ein2b* with KAR_1, AVG and $AgNO_3$; Arabidopsis *kai2*, *max2*, *d14*, *smax1 smxl2*, *acs7-1*, *ein2-1* with KAR_2 and ACC. No GR24.<br>**Crosstalk:** not addressed.	The module is conserved, but its genetic support differs for each species due to the availability of mutants in the ethylene pathway.	Carbonnel et al., 2020a; Villaécija-Aguilar et al., 2022
Strigolactone production and exudation under N or P limitation (§4.1)	Sorghum, *Astragalus sinicus*, alfalfa, lettuce,	Root exudates and root tissue of hydroponic seedlings, collected at 24 h intervals after 7–10 d in the test medium. Endogenous SLs quantified by LC-MS/MS; no signalling	None.	**n.a.:** nutrient omission from hydroponic medium only. No mutants, no receptor material and no analogues, so no branch can be assigned.<br>**Crosstalk:** not addressed.	The P response is conserved across all species tested. The N response is divergent: exudation rose in sorghum, Chinese milk vetch, lettuce,	Yoneyama et al., 2007, 2012

Process / output (section)	Species	Tissue, stage and conditions, main results	Biosynthetic, receptor and downstream components tested as knockout/down	Evidence class and tools, evidence of crosstalk	Conserved, species-specific or divergent	Reference
	marigold, tomato, wheat	component examined. Low nutrients generally increase SL production and exudation, with nuances across species and type of nutrient.			marigold and wheat, but not in alfalfa or tomato. A legume versus non-legume explanation was tested and rejected. Later, SL induction by low N was found in tomato (Marro et al., 2022)	
Convergence of PSR and KAI2-dependent signalling on NSP1–NSP2–SLR1 under low Pi (§4.1)	Rice	Roots sampled 7 d after transplanting; separate hydroponic Pi-starvation series. Gain-of-function in a null background: *PHR2* overexpression in *d14l*. Loss-of-function in a loss-of-function background: *smax1* in *phr123* triple mutant. PHR2 alone is used on the gain-of-function side because it is the most broadly root-expressed of the four rice PHRs, whereas PHR1, PHR2 and PHR3 are redundant and must all be removed for loss of function.<br>Readouts by RT-qPCR and RNA-seq. Transcripts only; no SL quantification and no protein-level data. The discovered path is PHR2→ *NSP1*/*NSP2*→ NSP1–NSP2–SLR1→ SL biosynthetic and Myc-perception genes. PHR2 and NSP2 bind the *D17* promoter directly.	PHR2 (and *phr1 phr2 phr3*), NSP1, NSP2, SLR1, D14L, SMAX1; SL biosynthetic targets D27, D17, D10, Os900 and Os1400.	**B:** cross-pathway epistasis, in both directions and with two designs. PHR2 overexpression assayed in the *d14l* background, and *smax1* assayed in the *phr1 phr2 phr3* background, so each pathway is removed while the other is activated.<br>**Crosstalk:** biosynthetic regulation. KAI2 (and PHR2) inputs converge on NSP1–NSP2 to activate SL biosynthesis genes.	Shown in rice. The two inputs are interdependent rather than additive: neither *PHR2* overexpression in *d14l* nor *smax1* in *phr123* triple mutant drives full KERG expression.	Yuan et al., 2023; Hong et al., 2026
N and P uptake, root dry weight and root-to-shoot ratio (§4.1)	Tomato	Whole plant across the life cycle; smoke water or 2 µM KAR(the metabolite is not declared in the paper) sprayed weekly from one month; 25/18 °C versus 18/12 °C with full Hoagland supply, so uptake chemical -limited .N, P and K by acid digestion, with gas exchange and antioxidant assays.	None in this experiment; the KAI2-, MAX1- and SnRK2.5-silenced lines from the same study are listed in the §4.3 entry	**C, E:** KAR and smoke water.<br>**Crosstalk** not addressed.	Tomato-specific as demonstrated, and conditional on the stress. Uptake and yield gains appear only at 18/12 °C; at 25/18 °C the same treatment lowers photosynthesis with no yield gain.	Liu et al., 2023
Presymbiotic transcriptional competence for AM symbiosis, gated by SMAX1 (§4.1)	Rice	Roots at 0–24 hpt with spore exudate, 1–7 dpi before fungal contact, and non-inoculated roots at 3 weeks. Colonisation scored at 4–7 wpi. *d14l* and *d3* show neither the transcriptional response to germinated spore exudate nor colonisation, whereas *d14* is unaffected; loss of *SMAX1* restores colonisation in both backgrounds and raises symbiosis and SL biosynthesis transcripts in the absence of fungus. GR24$^{ent\text{-}5DS}$, but	D14, D14L, D3, SMAX1, DMI3	**A:** receptor and repressor mutants combined with GR24$^{ent\text{-}5DS}$, GR24^{5DS}, KAR$_1$.<br>**Crosstalk:** biosynthetic regulation. This happens at the transcriptional level: SMAX1 represses the NSP1–NSP2–SLR1 complex, so KAI2-branch activation sets the transcription of SL biosynthesis genes upstream of the SL branch.	Demonstrated in rice, where the receptor requirement, the SMAX1 gate and the restoration of colonisation in double mutants are all established. Equivalent receptor-level and target-level genetic material has not been combined in another angiosperm.	Gutjahr et al., 2015; Choi et al., 2020; Hong et al., 2026

Process / output (section)	Species	Tissue, stage and conditions, main results	Biosynthetic, receptor and downstream components tested as knockout/down	Evidence class and tools, evidence of crosstalk	Conserved, species-specific or divergent	Reference
		not GR24^{5DS}, reproduces the AMF-induced gene set, and the response is lost in *d14l* and in the degradation-resistant *smax1-D*. KAR_1 at 10 µM acts likewise, although an earlier study reported no root transcriptional or colonisation response to KAR_1. *dmi3 smax1* is uncolonized, so SMAX1 removal is necessary but not sufficient. The discovered path is KL–D14L–D3→ SMAX1 degradation→ de-repression of NSP1–NSP2–SLR1, CSSP components and the SL pathway.				
Ethylene input into the SMAX1–SL–AM module (§4.1)	*Lotus japonicus*	Roots; ethylene acts mainly in the epidermis, shown with tissue-specific promoters. The discovered path is Ethylene→ EIN2→ SMAX1 accumulation→ repression of SL biosynthesis and Common Sym genes→ less colonisation. SMAX1 accumulation is KAI2-independent but reversed by KAR_1 in a KAI2-dependent way.	KAI2, SMAX1, EIN2.	**A:** *smax1*, *ein2a ein2b*, *kai2a kai2b* and tissue-specific *EIN2B-CEND*; ACC, ethephon and AVG, with KAR_1 and GR24^{5DS} as rescue.<br>**Crosstalk:** biosynthetic regulation. Ethylene converges on SMAX1, which gates SL biosynthesis; KAR_1 reverses the accumulation in a KAI2-dependent way.	Split. The SMAX1–SL–AM module is conserved among AM hosts; the ethylene input to AM is shown only here. SMAX1 accumulation also occurs in *N. benthamiana* and Arabidopsis, so that step is likely general and the AM output host-restricted.	Das et al., 2025
Absence of KAI2-dependent activation of SL biosynthesis (§4.1)	Arabidopsis (non-mycorrhizal); *Marchantia paleacea* (AM host, liverwort)	Whole Arabidopsis seedlings on agar (7 d to 2 weeks); *M. paleacea* thalli. The tested path is KAI2→ SMAX1/SMXL2→ SL biosynthesis. In both species the signalling arm is intact under the conditions tested: *DLK2* is strongly induced in *smax1 smxl2*. The biosynthetic arm is not engaged: SL biosynthesis does not appear to be activated and MeCLA is unchanged in *smax1 smxl2* relative to the *wt*. The same occurs in *M. paleacea.* This contrasts with rice, where the SMAX1→ SL biosynthesis link is established (Choi et al., 2020; Hong et al., 2026), and with *L. japonicus* (Das et al., 2025)	Arabidopsis: D14, KAI2, SMAX1, SMXL2<br>M. *palacea:* KAI2a, SMAX1.	**A:** *kai2-4*, *smax1*, *smxl2*, *smax1 smxl2*, *atd14-2*; *Mpasmxl* and *Mpakai2a* CRISPR lines; stereopure (−)-GR5; SLs quantified by LC-MS/MS.<br>**Crosstalk:** parallel activity without demonstrated molecular interaction. Crosstalk was tested directly, with negative results: KAI2 signalling does not activate SL biosynthesis in either species despite intact signalling output.	Divergent, and not simply host versus non-host. The link from KAI2 signalling to the activation of SL biosynthesis is present in mycorrhizal angiosperms, yet absent both in Arabidopsis, which is not an AM host, and in *M. paleacea*, which is. It was therefore probably acquired later than AM symbiosis itself. What differs between species is the SMAX1→ SL biosynthesis link rather than the signalling output, which remains intact in both systems.	Mashiguchi et al., 2023
Root elongation, P allocation and redox responses to smoke water under P limitation (§4.1)	Rice	Hydroponic seedlings; root meristem, epidermis and whole roots for RNA-seq. Smoke water enlarges the meristem, lowers H_2O_2 and MDA, and suppresses P-induced SL biosynthetic genes while leaving *D14*, *D14L*, *D3* and *D53* unchanged. Smoke water also shifted 1309 genes up and 1311	None.	**E,** at the weak end: smoke water only, no mutants and no parallel pure KARs. Smoke water also contains catechol, hydroquinone and antagonistic butenolides.<br>**Crosstalk:** proposed but untested interactions. Smoke water represses P-	Not assignable to the KAI2 pathway. Two phenotypes run opposite to established KAI2 outputs: root hairs are shortened, and SL biosynthetic genes are repressed. The gain is in P utilisation efficiency and allocation, not in uptake rate.	Pinit et al., 2023; Ariyakulkiat et al., 2026

Process / output (section)	Species	Tissue, stage and conditions, main results	Biosynthetic, receptor and downstream components tested as knockout/down	Evidence class and tools, evidence of crosstalk	Conserved, species-specific or divergent	Reference
		down under P deficiency, with "glutathione transferase activity" enriched among the up-regulated and "photosynthesis - light reaction" among the down-regulated set; it lowered root sucrose content and raised Fe accumulation		induced SL biosynthetic genes, with no receptor assignment.		
Contribution of the strigolactone metabolite to drought resistance (§4.2)	Arabidopsis; *Lotus japonicus*; tomato; rice	Rosettes and guard cells of 3- to 4-week-old Arabidopsis, with leaf and stem cuticle at 4 weeks; whole plants at the vegetative stage in the crop species. In Arabidopsis both SL-deficient and *d14* plants are drought-sensitive, and the defect lies in stomatal responsiveness rather than in ABA content, which is unchanged. In *Lotus* and tomato only the biosynthetic level has been tested. The same components operate in rice but with the opposite polarity.	Arabidopsis: MAX1, MAX3, MAX4, MAX2,D14<br>Lotus: CCD7<br>Tomato: CCD7<br>Rice: D10, D17, D27, D3 (and D27 overexpression)	**A, B:** SL biosynthetic mutants and *d14* alleles in Arabidopsis; *LjCCD7* RNAi with *rac*-GR24 rescue in *Lotus*; SL-depleted with reciprocal grafting in tomato; SL biosynthetic and signalling mutants in rice.<br>**Crosstalk:** proposed but untested interactions; additive activity without demonstrated molecular interaction.	Conserved in the dicots examined, with rice as a clear exception. In Arabidopsis the contribution of the metabolite is settled, because both biosynthetic mutants and *d14* lose drought resistance. *Lotus* and tomato agree at the biosynthetic level, although D14 has not been tested in either under drought. Rice runs the other way: SL-deficient and SL-insensitive mutants, with the exception of *d27*, are more drought-resistant than the wt, indicating a different wiring onto ABA metabolism rather than a weaker SL contribution. The only discordant report within Arabidopsis is Bu et al. (2014), where *max1*, *max3* and *max4* were indistinguishable from the wt, but was not confirmed since.	Bu et al., 2014; Liu et al., 2015; Visentin et al., 2016; Ha et al 2014; Haider et al., 2018; Li et al. 2020
Division of labour between D14 and KAI2 in drought acclimation (§4.2)	Arabidopsis	Rosettes and guard cells of 21- to 28-day-old plants; siliques and stems for wax crystals, ABA quantification, TEM/SEM. The outcomes are shared between the pathways: both receptors promote ABA-induced closure by increased sensitivity, *CYP707A3* (ABA catabolism) is also misregulated, bearing on ABA homeostasis rather than responsiveness. Differential: D14 is the larger contributor to anthocyanin biosynthesis, KAI2 to membrane integrity. The cuticle arm of this division of labour was established earlier and is reported in §3.2, not here.	D14, KAI2.	**B:** *d14-1*, *d14-2*, *kai2-2*, *d14-2 kai2-2*; survival assays, and a microarray of *d14-1* compared against the published *kai2-2* transcriptome in Li et al. (2017) (GEO GSE90622), so this row and §3.2 do not rest on independent transcriptomic evidence.<br>**Crosstalk:** additive activity without demonstrated molecular interaction. This is seen at the output level: the receptors act additively on survival and converge on ABA-induced stomatal closure.	Arabidopsis only, untested in other species. KAI2 and D14 act additively, with apparently stronger contribution by KAI2	Li et al., 2020

Process / output (section)	Species	Tissue, stage and conditions, main results	Biosynthetic, receptor and downstream components tested as knockout/down	Evidence class and tools, evidence of crosstalk	Conserved, species-specific or divergent	Reference
Direct induction of stomatal closure by GR24 analogues, and its relation to ABA (§4.2)	Arabidopsis; *Vicia faba*; tomato	Epidermal peels of fully expanded leaves of 4- to 6-week-old plants; *V. faba* strips; tomato leaves for porometry. In all three, short-term stomata closure is conserved. In Arabidopsis, the observed path is D14–MAX2→ H_2O_2 and NO→ SLAC1 since *rac*-GR24 fails to close *d14*, *max2*, *slac1*, *atrbohD atrbohF* and *nia1 nia2*, while H_2O_2 and SNP still close *d14*. The canonical ABA cascade is dispensable for guard-cell closure by GR24*. In tomato, less intense ABA sensing in SL-depleted plants likely operates also via miR156.	Arabidopsis: D14, MAX2, SLAC1, RBODH, NIA.<br>Faba bean: none.<br>Tomato: CCD7.	**B, C, D:** GR24*, with ABA-deficient panels and *slac1*, *atrboh* and *nia* alleles (Lv, Zhang). $GR24^{5DS}$ and SL-depleted plants but no receptor mutant (Visentin).<br>**Crosstalk:** not addressed. Closure by GR24* is lost in *d14*, but the KAI2-active enantiomer within *rac*-GR24 was never controlled for. No KAI2-related component examined directly.	The response is conserved in all three species at comparable concentrations, but pathway assignment is patchy: a KAI2 contribution has never been excluded (Lv; Zhang) although the response to *rac*-GR24 is blocked in *d14*. The tomato work used no receptor mutant, but SL-specific stereoisomer and knockdown of a biosynthetic gene (Visentin)	Lv et al., 2018; Zhang et al., 2018; Visentin et al., 2020
D14 targets SMAX1 for degradation under GR24 and osmotic stress (§4.2)	Arabidopsis; *Nicotiana benthamiana*; yeast for biochemistry	Hypocotyls of 5-d-old seedlings; axillary buds and rosette branches of adult plants; for the osmotic-stress work, seedlings grown on 300 mM mannitol for 14 days and scored at 21 d, with SMAX1 reporter degradation followed separately over 12 h. Under mannitol SMAX1 is degraded within 8 h in wt and *kai2* but not in *d14*, *max2* or *max3*, so the trigger is endogenous SL rather than an applied analogue. The outlined path is D14–MAX2→ SMAX1 and SMXL2, alongside SMXL6/7/8.		**A, B:** respectively for the ligand and the osmotic-stress work, which used no treatment. Receptor and target mutants in higher-order combinations with KAR_2, *rac*-GR24 and pure isomers; reporters in *d14a d14b Nicotiana*, yeast two-hybrid and FRET-APB.<br>**Crosstalk:** direct pathway cross-regulation. This is demonstrated at the target level and shown to be physiologically relevant: D14 degrades SMAX1, a KAI2-branch target, under applied $GR24^{5DS}$ and also under osmotic stress without any applied compound. The promiscuity is one-way: no evidence that KAI2 targets SMXL6/7/8. Apparently not merely a vestige of D14 evolution from KAI2.	Arabidopsis only. Sets a limit on branch insulation: target assignment does not necessarily hold under all conditions.	Li et al., 2022
Drought priming and the stomatal after-effect through the SL–miR156 module (§4.2)	Tomato	Apical leaves and guard cells of 4-week-old plants during dehydration and recovery; buds and roots of 10-week-old plants for stem-loop qRT-PCR, UPLC-MS/MS for ABA, porometry. $GR24^{5DS}$ raises miR156 from 6 h; drought fails to induce it in *CCD7*-silenced leaves. Closure occurs within 2 h, before miR156 rises, so the module accounts for the delayed ABA-dependent component and not the fast closure. miR156 overexpression lowers free ABA yet strengthens closure in response to applied ABA due to higher sensitivity.	CCD7, miR156-OE.	**A:** $GR24^{5DS}$, *CCD7* knockdown, miR156 overexpressor. No receptor mutant.<br>**Crosstalk:** not addressed. KAI2/KARs untested.	Tomato only. The role of SLs in the stomatal after-effect of drought later confirmed in Arabidopsis (Korwin Krukowski et al., 2022).	Visentin et al., 2020; Korwin Krukowski et al.,2022

Process / output (section)	Species	Tissue, stage and conditions, main results	Biosynthetic, receptor and downstream components tested as knockout/down	Evidence class and tools, evidence of crosstalk	Conserved, species-specific or divergent	Reference
Trainability of drought memory under repeated stress (§4.2)	Tomato	Leaves and guard cells of 4-week-old plants through two dehydration and rewatering cycles for wt and SL-depleted, with leaf RNA-seq at pre-stress and both recovery points, plus $GR24^{5DS}$-treated. Wt shows a stronger after-effect in the second cycle; about half of drought-trainable genes lose trainability without SLs. SL-dependent clusters are enriched in HSPs and *HSFA2* and *ROF1* orthologues.	CCD7.	**A:** $GR24^{5DS}$, *CCD7* knockdown. **Crosstalk:** not addressed. KAI2/KARs untested.	Tomato only. Sustained SL-dependent expression during recovery is known in Arabidopsis (Korwin Krukowski et al., 2022), but trainability by repeated stress has not been tested yet.	Visentin et al., 2025; Korwin Krukowski et al.,2022
Salt tolerance: germination, ion homeostasis, oxidative damage and hormone crosstalk (§4.2)	Arabidopsis; *Brassica napus*	Seeds scored up to 13 d on 0–300 mM NaCl; rosette shoots at 21 d after 14 d of NaCl; *B. napus* seedlings after 7 d, with RNA-seq at 12 h. Both branches act positively through MAX2. *max2*, *max3* and *max4* all show reduced survival. *kai2* shoots hold more Na^+ and less K^+, with lower *SOS1–SOS3* and *HKT1;1* and reduced antioxidant capacity. ABA biosynthetic transcripts are unchanged whereas signalling and catabolism are lower.	Arabidopsis: MAX3, MAX4, KAI2, MAX2 *B. napus*: none (chemical treatment only)	Arabidopsis: **B, D** from *htl-3*, *max3*, *max4* and *max2*, *rac*-GR24 *B. napus*: **D** from GR24*. No KAR treatment in any study. **Crosstalk:** proposed but untested interactions. Both branches promote salt tolerance through MAX2, yet no study combined or separated them directly. The SL side used GR24* and biosynthetic mutants. Neither SL content nor any SL-signalling output was measured (SMAX1 and the SMXLs were not tested), so reduced SL signalling in *kai2* is inferred from transcript levels of SL biosynthesis and signalling genes alone.	Genetic evidence is confined to Arabidopsis. HTL (HYPOSENSITIVE TO LIGHT) is a synonym of KAI2, not a paralogue, so *htl-3* is an Arabidopsis *kai2* allele. No receptor mutant was used in *B. napus*, where the work was GR24* treatment only	Ma et al., 2017; Ha et al., 2014; Li et al., 2017; Mostofa et al., 2023
Germination, seedling establishment and redox homeostasis under salt and osmotic stress (§4.2)	*Sapium sebiferum* (*Triadica sebifera*)	Seeds on 100–200 mM NaCl or 100–300 mM mannitol, scored 10 d after imbibition; seedlings stressed from 12 d with 150 mM NaCl or 200 mM mannitol, growth scored after 12–15 d; roots at 3, 6 and 9 d for redox assays and at 4, 8 and 12 h for transcripts, the latter under 200 mM NaCl rather than 150 mM; leaves at 6 d for GC-TOF-MS and for ABA. KAR_1 supplied at 1 nM continuously in the medium throughout. Scored outputs: germination, survival, taproot/lateral root number, shoot length, biomass, F_v/F_m, H_2O_2, MDA, electrolyte leakage, SOD/POD/CAT/ APX activity, leaf ABA content + transcripts of *NCED3*, *NCED9*, *SnRK2.3*, *SnRK2.6*, *ABI3*, *ABI5*, *SOS1*, *DREB2A*, *WRKY33*, *ERF6*. Putative *KAI2*, *MAX2*, *DLK2* and	None.	**C:** KAR_1. **Crosstalk:** not addressed.	*S. sebiferum* only. The seedling-level physiology matches KAR_1 reports in wheat and in creeping bentgrass, so that output may be conserved, but the germination result diverges from Arabidopsis, where KAR–KAI2 signalling restrains germination under conditions unfavourable for seedling establishment (Wang et al., 2018). Branch-specific mutants needed for final attribution.	Shah et al., 2020

Process / output (section)	Species	Tissue, stage and conditions, main results	Biosynthetic, receptor and downstream components tested as knockout/down	Evidence class and tools, evidence of crosstalk	Conserved, species-specific or divergent	Reference
		KUF1 transcripts rise and *SMAX1* falls under stress, KAR_1 amplifies each change.				
Salt tolerance: ion homeostasis, redox balance and metabolite reprogramming after foliar KAR_1 (§4.2)	Creeping bentgrass (*Agrostis stolonifera*)	Leaves of hydroponic plants after 30 d of acclimation; 100 nM KAR_1 sprayed daily for 3 d before stress and weekly thereafter; 200 mM NaCl reached in 50 mM daily steps and held for 21 d; single harvest at 21 d. Outputs: shoot FW, DW, RWC, chlorophyll, proline, electrolyte leakage, MDA, H_2O_2, SOD, CAT, APX activity, Na^+, K^+ and Mg^{2+} by ICP-OES, untargeted LC-MS metabolome. KAR_1 raised shoot biomass, leaf RWC, total chlorophyll and chlorophyll *a*, proline and antioxidant enzyme activity under salt, while lowering electrolyte leakage, MDA and H_2O_2; it raised leaf K^+ and Mg^{2+} and lowered the Na^+/K^+ ratio. Metabolomics linked the effect to accumulation of specific amino acids for osmotic adjustment and to the α-linolenic acid and phenylpropanoid biosynthesis pathways. KAR_1 also raised shoot FW, total chlorophyll and SOD activity in the absence of salt, so the response is not stress-conditional.	None.	**C:** KAR_1.<br>**Crosstalk:** not addressed.	Creeping bentgrass only, and the first report in a cool-season turfgrass. The direction agrees with KAR_1 studies in wheat and *S. sebiferum* and with the salt-sensitive *kai2* phenotype in Arabidopsis (Mostofa et al., 2023), so the physiological output is plausibly conserved. Attribution to KAI2 is untested here.	Wang et al., 2025
Heat and cold tolerance: HSPs, CBF1 and antioxidant capacity, downstream of ABA (§4.3)	Tomato	Roots of 5-week-old plants $GR24^{5DS}$ 3μM to roots, 24 h before stress; leaves of the same plants for tolerance phenotypes and stress-responsive transcripts after 48 h at 42 °C or 7 d at 4 °C. ABA and SLs quantified by LC-MS/MS (after 3 h at 42 °C, 6 h at 4 °C). *CCD7*, *CCD8* and *MAX1* transcripts rise in roots within 3–6 h of heat or cold, as does root solanacol (orobanchol/ didehydro-orobanchol unchanged). *ccd7* plants show lower Φ_PSII, heat-induced HSP70 and cold-induced *CBF1*, higher electrolyte leakage; $GR24^{5DS}$ reverses these effects, raises leaf ABA under stress. In *notabilis*, $GR24^{5DS}$ no longer effective.	CCD7, CCD8, MAX1, MAX2, NCED1	**A:** $GR24^{5DS}$ with a CRISPR frameshift *ccd7* allele retaining 37–42% of root SLs, and VIGS knockdown of *CCD7*, *CCD8*, *MAX1* and *MAX2* (72–80% reduction); $GR24^{5DS}$ also applied in the ABA-deficient *notabilis* (*nced1*) background.<br>**Crosstalk:** not addressed.	Tomato only. The biosynthetic direction is stress-specific rather than species-specific: heat and cold raise root SL biosynthesis, whereas drought and salinity repress it in the same species (Visentin et al., 2016). The output is ABA-dependent, unlike the ABA-independent stomata closure reported in Arabidopsis (Lv et al., 2018; Zhang et al., 2018L).	Chi et al., 2021
Cold acclimation: CBF induction,	Tomato	Leaves of one-month-old seedlings, 6 h pre-treatment then 6 d at 10/5 °C	KAI2, MAX1, SnRK2.5.	**A** for the KAR arm (KAR* with *KAI2* silencing), **B, C** for the SL arm (GR24*	Tomato only. KAR still confers cold tolerance in *MAX1*-	Liu et al., 2023

**Process / output (section)**	**Species**	**Tissue, stage and conditions, main results**	**Biosynthetic, receptor and downstream components tested as knockout/down**	**Evidence class and tools, evidence of crosstalk**	**Conserved, species-specific or divergent**	**Reference**
ABA sensitivity and stomatal control (§4.3)		*KAI2*-silenced plants are cold-sensitive and are not rescued by smoke water or KAR. KAR raises *KAI2*, *D14*, *MAX2.1*, *MAX2.2* and *MAX1* but not *CCD7* or *CCD8*, and none of this occurs when *KAI2* is silenced. Silencing *KAI2*, *MAX1* or *SnRK2.5* lowers *CBF1–CBF3*.		with *MAX1* silencing), **E** for smoke water. **Crosstalk:** biosynthetic regulation, direct pathway cross-regulation and convergence on a common hormonal target. Evidence exists at the transcript level: KAR* raises *D14*, *MAX1* and *MAX2.1/2.2* KAI2-dependently, yet KAR-induced tolerance survives *MAX1* silencing, so the branches converge on *CBF* induction without a strict biosynthetic relay.	silenced plants, so the KAI2 branch is not strictly routed through *MAX1* despite the paper's summary. The ABA direction opposes Chi et al. (2021) in the same species under the same stress.	
Inhibition of mesocotyl elongation in darkness (§4.4)	Rice	Mesocotyls of 7-d-old dark-grown seedlings; tillers counted and height measured at heading; calli and protoplasts for protein stability; immunoblot, ubiquitination, Co-IP and RNA-seq. The outlined paths are D14L–D3–SMAX1, parallel to D14–D3–D53. D14L binds SMAX1 and D3 but not D53; D14 does not bind SMAX1. SMAX1 accumulates in *d14l* and *d3* but not *d14*.	D10, D17, D14, D14L, D3, SMAX1, D53.	**A:** $GR24^{5DS}$ and $GR24^{ent\text{-}5DS}$, KAR_1 and KAR_2 on *d14l, smax1*, *d14*, *d3*, *d10*, *d17*, *d53*. The authors note that neither KAR_1 nor $GR24^{ent\text{-}5DS}$ enhances the D14L–SMAX1 interaction *in vitro*. **Crosstalk:** direct cross-regulation, but partial. Loss of SMAX1 also rescues *d3*, *d14* and *d10*, so the branches are not fully separable, although D14 does not bind SMAX1.	The module architecture is conserved with Arabidopsis hypocotyls.	Zheng et al., 2020
Seed germination and dormancy under mild osmotic stress, salinity and elevated temperature (§4.2,5)	Arabidopsis	Imbibed seeds after-ripened for about 3 months, scored daily from 7 to 14 d, with freshly harvested dormant batches in some experiments; seeds imbibed 48 h then treated a further 24 h for transcripts; 14-day-old seedlings for hypocotyl elongation; gynoecia and siliques for *KAI2* expression. KAR_2 at 1 µM with a 0.2–2.0 µM dose series, and GA_3, KNO_3 and ABA as comparators; NaCl and mannitol to 100 mM, sugars, 27 and 30 °C KAI2 acting with MAX2; D14 dispensable under osmotic stress but untested at high T. *KAI2* is expressed throughout seed development. *DLK2* and *KUF1* remain KAR_2-inducible under stress and hypocotyl inhibition is unchanged, so signalling itself is intact; *WRKY33*, *ERF5* and *DREB2A* are KAR_2-responsive only under mild osmotic stress. ABA biosynthesis, signalling and response genes and *DOG1* are unaffected by KAR_2, although *kai2* seeds are more sensitive to exogenous ABA than the wt.	D14, KAI2, MAX2, ABA2, ABA3	**A:** *kai2*, *d14* and *max2-7* in Ler for all germination assays, with Col-0 lines (*kai2*, *d14*, *max2-1*) for hypocotyl elongation and ABA sensitivity, + *aba3*, *aba2*; KAR_2. No GR24 isomer used. **Crosstalk:** proposed but untested interactions. Crosstalk is unresolved for the heat arm: *d14* behaves as the wt under osmolytes and salt, so that output is KAI2–MAX2 specific. D14 was not tested in the heat experiments, where SLs have been reported to relieve thermo-inhibition (Toh et al., 2012).	Arabidopsis only. KAI2 confers germinability and tolerance to osmotic and thermal stress, while the direction of the KAR_2 effect is stress-conditional, from slight stimulation in unstressed dormant seed to further inhibition above 20 mM NaCl. This contrasts with the germination-stimulant role of KARs in fire-followers, which has not been tested under comparable stress; Arabidopsis is not itself a fire ephemeral. The effect is also detectable only under restrictive conditions, since Col-0 has little dormancy and reduced ABA sensitivity, and standard nitrate- and sucrose-containing media mask the response.	Wang et al., 2018<br>Toh et al., 2012

Process / output (section)	Species	Tissue, stage and conditions, main results	Biosynthetic, receptor and downstream components tested as knockout/down	Evidence class and tools, evidence of crosstalk	Conserved, species-specific or divergent	Reference
Vegetative growth, seedling vigour and yield traits after smoke water or KAR_1 (§5)	Tomato, okra, cucumber, gladiolus, scotch marigold, *Mentha arvensis*	Okra and tomato seedlings sprayed to run-off four times between 8 and 20 d after sowing and harvested at 28 d (Kulkarni); seeds of cucumber, tomato and scotch marigold, and gladiolus cormels soaked 24 h before sowing, germination scored at 21 d and seedlings harvested after one month (Elsadek and Yousef); mint raised from suckers, foliage sprayed five times at 5-d intervals and sampled 7 d after the last spray, 90 d after planting (Singh). In mint a KAI2–MAX2–SMAX1 route is proposed in a summary scheme with no genetic or biochemical support. In okra, pure KAR_1 is not equivalent to the mixture: it had no effect at the single dose tested and lowered root DW; in mint smoke water outperformed KAR_1 for most traits. In the four horticultural crops the active smoke waters lowered seed ABA and raised α-amylase activity, and raised shoot N and P	None.	**C, D:** complex mixtures throughout, with the source material differing between studies (*Themeda triandra* at 1:500 v/v; five species compared, of which only white willow and lemon eucalyptus were active; wheat straw at 1:125 to 1:1000 v/v). Pure KAR_1 was applied in parallel in two of the three, as 0.1 µM smoke-isolated butenolide and as 10^{-9} to 10^{-6} M karrikinolide. **Crosstalk** not addressed.	Growth promotion is reproducible across distant crops, but this is convergence of an applied phenotype rather than evidence of a conserved pathway. Outcomes depend on source material, dose and light: rice-straw and rosemary smoke water was inert where white willow and lemon eucalyptus was active; the dose response is non-monotonic, the highest concentrations being weaker than intermediate ones; and the stimulation was lost in darkness in tomato, gladiolus and scotch marigold, though not in cucumber.	Kulkarni et al., 2007; Elsadek and Yousef, 2019; Singh et al., 2023

Evidence classes: A, pathway-specific mutants and branch-selective pure ligand (a stereopure GR24 isomer/natural SL, or pure KAR with *kai2/d14l* or *d14*); **B**, pathway-specific mutants only; **C**, selective ligands only (no pathway-specific mutants); **D**, *max2*, *d3* or *rac*-GR24 only, which cannot separate the branches; **E**, smoke water without receptor-specific mutants; **n.a.**, no findings bearing on assignment to either branch. For mutants/transgenic lines, knockdown rather than knockout is stated where relevant. **GR24*** stands for unspecified stereochemistry, but most likely *rac*-GR24: **KAR*** when the specific metabolite is not declared. Hpt: hours post treatment. Dpg: days post germination.

References

Ariyakulkiat, L., Pinit, S., and Chaiwanon, J. (2026). Smoke water promotes root meristem activity and cell elongation but inhibits root hair growth under phosphorus deficiency in rice. *S. Afr. J. Bot.* 191, 12–19. doi: 10.1016/j.sajb.2026.02.012

Bu, Q., Lv, T., Shen, H., Luong, P., Wang, J., Wang, Z., et al. (2014). Regulation of drought tolerance by the F-Box protein MAX2 in Arabidopsis. *Plant Physiol.* 164, 424–439. doi: 10.1104/pp.113.226837

Carbonnel, S., Das, D., Varshney, K., Kolodziej, M. C., Villaécija-Aguilar, J. A., and Gutjahr, C. (2020a). The karrikin signaling regulator SMAX1 controls Lotus japonicus root and root hair development by suppressing ethylene biosynthesis. *Proc. Natl. Acad. Sci. U.S.A.* 117, 21757–21765. doi: 10.1073/pnas.2006111117

Chi, C., Xu, X., Wang, M., Zhang, H., Fang, P., Zhou, J., et al. (2021). Strigolactones positively regulate abscisic acid-dependent heat and cold tolerance in tomato. *Hortic. Res.* 8, 237. doi: 10.1038/s41438-021-00668-y

Choi, J., Lee, T., Cho, J., Servante, E. K., Pucker, B., Summers, W., et al. (2020). The negative regulator SMAX1 controls mycorrhizal symbiosis and strigolactone biosynthesis in rice. *Nat. Commun.* 11, 2114. doi: 10.1038/s41467-020-16021-1

Das, D., Varshney, K., Ogawa, S., Torabi, S., Hüttl, R., Nelson, D. C., et al. (2025). Ethylene promotes SMAX1 accumulation to inhibit arbuscular mycorrhiza symbiosis. *Nat. Commun.* 16, 2025. doi: 10.1038/s41467-025-57222-w

Elsadek, M. A., and Yousef, E. A. A. (2019). Smoke-water enhances germination and seedling growth of four horticultural crops. *Plants* 8, 104. doi: 10.3390/plants8040104

Gomez-Roldan, V., Fermas, S., Brewer, P. B., Puech-Pagès, V., Dun, E. A., Pillot, J.-P., et al. (2008). Strigolactone inhibition of shoot branching. *Nature* 455, 189–194. doi: 10.1038/nature07271

Gutjahr, C., Gobbato, E., Choi, J., Riemann, M., Johnston, M. G., Summers, W., et al. (2015). Rice perception of symbiotic arbuscular mycorrhizal fungi requires the karrikin receptor complex. *Science* 350, 1521–1524. doi: 10.1126/science.aac9715

Ha, C. V., Leyva-González, M. A., Osakabe, Y., Tran, U. T., Nishiyama, R., Watanabe, Y., et al. (2014). Positive regulatory role of strigolactone in plant responses to drought and salt stress. *Proc. Natl. Acad. Sci. U.S.A.* 111, 851–856. doi: 10.1073/pnas.1322135111

Haider, I., Andreo-Jimenez, B., Bruno, M., Bimbo, A., Floková, K., Abuauf, H., et al. (2018). The interaction of strigolactones with abscisic acid during the drought response in rice. *J. Exp. Bot.* 69, 2403–2414. doi: 10.1093/jxb/ery089

Hong, K., Yang, X., Tan, Y., Liu, Y., Xia, Q., Zeng, L., et al. (2026). Presymbiotic activation of karrikin signaling creates a permissive state for arbuscular mycorrhizal symbiosis by derepressing the NSP1–NSP2–SLR1 transcriptional complex in rice. *Mol. Plant* 19, 844–867. doi: 10.1016 j.molp.2026.01.014

Korwin Krukowski, P., Visentin, I., Russo, G., Minerdi, D., Bendahmane, A., Schubert, A., et al. (2022). Transcriptome analysis points to BES1 as a transducer of strigolactone effects on drought memory in Arabidopsis thaliana. *Plant Cell Physiol.* 63, 1873–1889. doi: 10.1093/pcp/pcac058

Kulkarni, M. G., Ascough, G. D., and Van Staden, J. (2007). Effects of foliar applications of smoke-water and a smoke-isolated butenolide on seedling growth of okra and tomato. *Hortic. Sci.* 42, 179–182. doi: 10.21273/HORTSCI.42.1.179

Li, Q., Martín-Fontecha, E. S., Khosla, A., White, A. R. F., Chang, S., Cubas, P., et al. (2022). The strigolactone receptor D14 targets SMAX1 for degradation in response to GR24 treatment and osmotic stress. *Plant Commun.* 3, 100303. doi: 10.1016/j.xplc.2022.100303

Li, W., Nguyen, K. H., Chu, H. D., Ha, C. V., Watanabe, Y., Osakabe, Y., et al. (2017). The karrikin receptor KAI2 promotes drought resistance in Arabidopsis thaliana. *PLoS Genet.* 13, e1007076. doi: 10.1371/journal.pgen.1007076

Li, W., Nguyen, K. H., Chu, H. D., Watanabe, Y., Osakabe, Y., Sato, M., et al. (2020). Comparative functional analyses of DWARF14 and KARRIKIN INSENSITIVE 2 in drought adaptation of Arabidopsis thaliana. *Plant J.* 103, 111–127. doi: 10.1111/tpj.14712

Liu, J., He, H., Vitali, M., Visentin, I., Charnikhova, T., Haider, I., et al. (2015). Osmotic stress represses strigolactone biosynthesis in Lotus japonicus roots: exploring the interaction between strigolactones and ABA under abiotic stress. *Planta* 241, 1435–1451. doi: 10.1007/s00425-015-2266-8

Liu, M., Shan, Q., Ding, E., Gu, T., and Gong, B. (2023). Karrikin increases tomato cold tolerance via strigolactone and the abscisic acid signaling network. *Plant Sci.* 332, 111720. doi: 10.1016/j.plantsci.2023.111720

Lv, S., Zhang, Y., Li, C., Liu, Z., Yang, N., Pan, L., et al. (2018). Strigolactone-triggered stomatal closure requires hydrogen peroxide synthesis and nitric oxide production in an abscisic acid-independent manner. *New Phytol.* 217, 290–304. doi: 10.1111/nph.14813

Ma, N., Hu, C., Wan, L., Hu, Q., Xiong, J., and Zhang, C. (2017). Strigolactones improve plant growth, photosynthesis, and alleviate oxidative stress under salinity in rapeseed (Brassica napus L.) by regulating gene expression. *Front. Plant Sci.* 8, 1671. doi: 10.3389/fpls.2017.01671

Marro, N., Lidoy, J., Chico, M. Á., Rial, C., García, J., Varela, R. M., et al. (2022). Strigolactones: new players in the nitrogen–phosphorus signalling interplay. *Plant Cell Environ.* 45, 512–527. doi: 10.1111/pce.14212

Mashiguchi, K., Morita, R., Tanaka, K., Kodama, K., Kameoka, H., Kyozuka, J., et al. (2023). Activation of strigolactone biosynthesis by the DWARF14-LIKE/KARRIKIN-INSENSITIVE2 pathway in mycorrhizal angiosperms, but not in Arabidopsis, a non-mycorrhizal plant. *Plant Cell Physiol.* 64, 1066–1078. doi: 10.1093/pcp/pcad079

Mostofa, M. G., Abdelrahman, M., Rahman, Md. M., Tran, C. D., Nguyen, K. H., Watanabe, Y., et al. (2023). Karrikin receptor KAI2 coordinates salt tolerance mechanisms in Arabidopsis thaliana. *Plant Cell Physiol.* 63, 1761–1763. doi: 10.1093/pcp/pcac148

Moturu, T. R., Thula, S., Singh, R. K., Nodzyński, T., Vařeková, R. S., Friml, J., et al. (2018). Molecular evolution and diversification of the SMXL gene family. *J. Exp. Bot.* 69, 2367–2378. doi: 10.1093/jxb/ery097

Pinit, S., Ariyakulkiat, L., and Chaiwanon, J. (2023). Rice straw-derived smoke water promotes rice root growth under phosphorus deficiency by modulating oxidative stress and photosynthetic gene expression. *Sci. Rep.* 13, 14802. doi: 10.1038/s41598-023-41987-5

Salem, M. A., Jamil, M., Wang, J. Y., and Al-Babili, S. (2025). Disruption of the karrikin receptor *DWARF 14-LIKE* (*D14L*) gene leads to distinct effects on root and shoot growth, and reprogramming of central metabolism in rice. *J. Exp. Bot.* 76, 4114–4128. doi: https://doi.org/10.1093/jxb/eraf201

Shah, F. A., Wei, X., Wang, Q., Liu, W., Wang, D., Yao, Y., et al. (2020). Karrikin improves osmotic and salt stress tolerance via the regulation of the redox homeostasis in the oil plant Sapium sebiferum. *Front. Plant Sci.* 11, 216. doi: 10.3389/fpls.2020.00216

Singh, S., Uddin, M., Chishti, A. S., Bhat, U. H., Singh, S., and Khan, M. M. A. (2023). Plant-derived smoke water and karrikinolide (KAR1) enhance physiological activities, essential oil yield and bioactive constituents of Mentha arvensis L. *Front. Plant Sci.* 14, 1129130. doi: 10.3389/fpls.2023.1129130

Soundappan, I., Bennett, T., Morffy, N., Liang, Y., Stanga, J. P., Abbas, A., et al. (2015). SMAX1-LIKE/D53 family members enable distinct MAX2-dependent responses to strigolactones and karrikins in Arabidopsis. *Plant Cell* 27, 3143–3159. doi: 10.1105/tpc.15.00562

Stanga, J. P., Morffy, N., and Nelson, D. C. (2016). Functional redundancy in the control of seedling growth by the karrikin signaling pathway. *Planta* 243, 1397–1406. doi: 10.1007/s00425-015-2458-2

Toh, S., Kamiya, Y., Kawakami, N., Nambara, E., McCourt, P., and Tsuchiya, Y. (2012). Thermoinhibition uncovers a role for strigolactones in Arabidopsis seed germination. *Plant Cell Physiol.* 53, 107–117. doi: 10.1093/pcp/pcr176

Umehara, M., Hanada, A., Yoshida, S., Akiyama, K., Arite, T., Takeda-Kamiya, N., et al. (2008). Inhibition of shoot branching by new terpenoid plant hormones. *Nature* 455, 195–200. doi: 10.1038/nature07272

Villaécija-Aguilar, J. A., Hamon-Josse, M., Carbonnel, S., Kretschmar, A., Schmidt, C., Dawid, C., et al. (2019). SMAX1/SMXL2 regulate root and root hair development downstream of KAI2-mediated signalling in Arabidopsis. *PLoS Genet.* 15, e1008327. doi: 10.1371/journal.pgen.1008327

Villaécija-Aguilar, J. A., Körösy, C., Maisch, L., Hamon-Josse, M., Petrich, A., Magosch, S., et al. (2022). KAI2 promotes Arabidopsis root hair elongation at low external phosphate by controlling local accumulation of AUX1 and PIN2. *Curr. Biol.* 32, 228-236.e3. doi: 10.1016/j.cub.2021.10.044

Visentin, I., Vitali, M., Ferrero, M., Zhang, Y., Ruyter-Spira, C., Novák, O., et al. (2016). Low levels of strigolactones in roots as a component of the systemic signal of drought stress in tomato. *New Phytol.* 212, 954–963. doi: 10.1111/nph.14190

Visentin, I., Pagliarani, C., Deva, E., Caracci, A., Turečková, V., Novák, O., et al. (2020). A novel strigolactone-miR156 module controls stomatal behaviour during drought recovery. *Plant Cell Environ.* 43, 1613–1624. doi: 10.1111/pce.13758

Visentin, I., Campo, E., Davydenko, D., Korwin Krukowski, P., Russo, G., Lovisolo, C., et al. (2025). Strigolactones affect the stomatal and transcriptomic memory of repeated drought stress in tomato. *Plant Stress* 15, 100740. doi: 10.1016/j.stress.2025.100740

Wang, L., Waters, M. T., and Smith, S. M. (2018). Karrikin-KAI2 signalling provides Arabidopsis seeds with tolerance to abiotic stress and inhibits germination under conditions unfavourable to seedling establishment. *New Phytol.* 219, 605–618. doi: 10.1111/nph.15192

Wang, L., Xu, Q., Yu, H., Ma, H., Li, X., Yang, J., et al. (2020). Strigolactone and karrikin signaling pathways elicit ubiquitination and proteolysis of SMXL2 to regulate hypocotyl elongation in Arabidopsis. *Plant Cell* 32, 2251–2270. doi: 10.1105/tpc.20.00140

Wang, Y., Han, Y., Zhu, M., Li, X., Liu, Y., Tan, Z., et al. (2025). Karrikin1 mitigates salt stress in creeping bentgrass (Agrostis stolonifera) via physiological regulation and metabolite reprogramming. *Plant Physiol. Biochem.* 229, 110800. doi: 10.1016/j.plaphy.2025.110800

Yoneyama, K., Xie, X., Kusumoto, D., Sekimoto, H., Sugimoto, Y., Takeuchi, Y., et al. (2007). Nitrogen deficiency as well as phosphorus deficiency in sorghum promotes the production and exudation of 5-deoxystrigol, the host recognition signal for arbuscular mycorrhizal fungi and root parasites. *Planta* 227, 125–132. doi: 10.1007/s00425-007-0600-5

Yoneyama, K., Xie, X., Kim, H. I., Kisugi, T., Nomura, T., Sekimoto, H., et al. (2012). How do nitrogen and phosphorus deficiencies affect strigolactone production and exudation? *Planta* 235, 1197–1207. doi: 10.1007/s00425-011-1568-8

Yuan, K., Zhang, H., Yu, C., Luo, N., Yan, J., Zheng, S., et al. (2023). Low phosphorus promotes NSP1–NSP2 heterodimerization to enhance strigolactone biosynthesis and regulate shoot and root architecture in rice. *Mol. Plant* 16, 1811–1831. doi: 10.1016/j.molp.2023.09.022

Zhang, Y., Lv, S., and Wang, G. (2018). Strigolactones are common regulators in induction of stomatal closure *in planta*. *Plant Signal. Behav.* 13, e1444322. doi: 10.1080/15592324.2018.1444322

Zheng, J., Hong, K., Zeng, L., Wang, L., Kang, S., Qu, M., et al. (2020). Karrikin signaling acts parallel to and additively with strigolactone signaling to regulate rice mesocotyl elongation in darkness. *Plant Cell* 32, 2780–2805. doi: 10.1105/tpc.20.00123
